# Supplementary material for: MiR-128-3p – a gray eminence of the human central nervous system
Source: Mol Ther Nucleic Acids. 2024 Feb 6;35(1):102141. doi: 10.1016/j.omtn.2024.102141 (PMC10899074; doi:10.1016/j.omtn.2024.102141)
Supplement: Document S1. Supplemental materials and methods, Figures S1 and S2, and Table S1 [file mmc1.pdf]

OMTN, Volume *35*

Supplemental information

MiR-128-3p – a gray eminence of the human central nervous system

Klaudia Kiel, Sylwia Katarzyna Król, Agnieszka Bronisz, and Jakub Godlewski

SUPPLEMENTAL MATERIALS AND METHODS

Databases and Data Selection

For the examination of miR-128 gene family expression in the human body and brain compartments, the RNA Tissue Atlas dataset was employed [1]. Briefly, data in the miRNATissueAtlas2 were generated using a stand-alone version of our web-based tool miRMaster [2, 3]. The most recent online version supports the same features as the stand-alone version, most notably the multi-species support [4]. We conducted all analyses using the standard parameter settings as in the online version. The following ncRNA databases are included in the analysis: miRBase version 22.1 [5], Ensembl ncRNA version 100 [6], RNACentral version 15 [7], GtRNAdb version 18.1 [8] and NONCODE version 5 [9]. Subsequently, miEAA - microRNA Enrichment and Annotation Analysis [10] results were incorporated for review, utilizing the Gene Ontology (GO) database (December 2023 edition) and Shine Go version 0.79 of the application [11].

The source code is accessible at https://github.com/iDEP-SDSU/idep/tree/master/shinyapps/go61. Current database files are available at https://doi.org/10.5281/zenodo.1451847.
All data included in the miRNATissueAtlas2 are freely accessible from the Gene Expression Omnibus (GSE163534).

MicroRNA Expression Analysis

Expression Mean, log-transformed, normalized values (>0.01 RKPM) from the RNA Tissue Atlas databases were utilized to define microRNA signatures based on the expression of mature transcripts from whole brain tissue and its compartments. Data collection adheres to all relevant laws, regulations, and policies for protecting human subjects (see Table S1).

Functional and Statistical Analysis

A hierarchical clustering tree summarizes the correlation among significant Pathways databases listed in the Figure Legend [12-18] for miR-128-3p target genes input (n=1225) selected based on Target Scan v8 [16]. All statistical operations were performed with GraphPad Prism 8 software/ excel, considering significance with a False Discovery Rate (FDR > 0.05) with corrected q-value < 0.01 and correlation r value = +/-0.5.

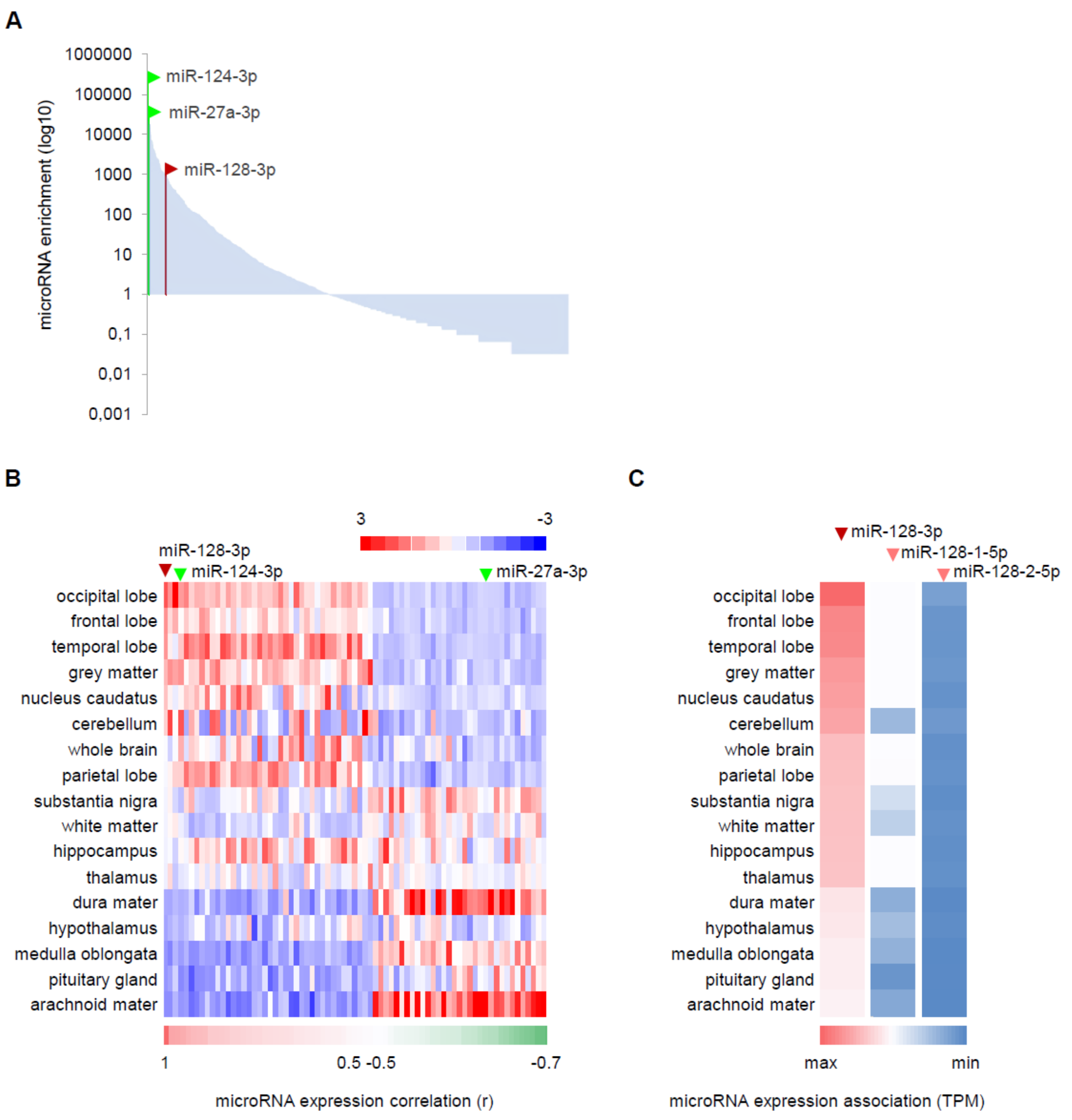

Figure S1. Brain tissue microRNAome overview.
A MicroRNAome enrichment in brain tissue is based on mean, log-transformed, normalized values (>0.01) downloaded from the RNA Tissue Atlas database (Table S1/Spreadsheet: 2 and 3). Mature microRNA n=1595.

B MiR-128-3p microRNAome expression correlation brain tissue compartments matrix is based on mean, log-transformed, normalized values (value > 0.01), downloaded from the RNA Tissue Atlas database and correlated with miR-128-3p with r value ≥ ±0.5. (Table S1/Spreadsheet: 4). Mature microRNA n=73.

C MiR-128-3p family mature microRNAs expression association heat map is based on mean, log-transformed, normalized values of miR-128-3p transcripts downloaded from the RNA Tissue Atlas database (Table S1/Spreadsheet: 5). Mature microRNA n=3. TPM - transcripts per kilobase million.

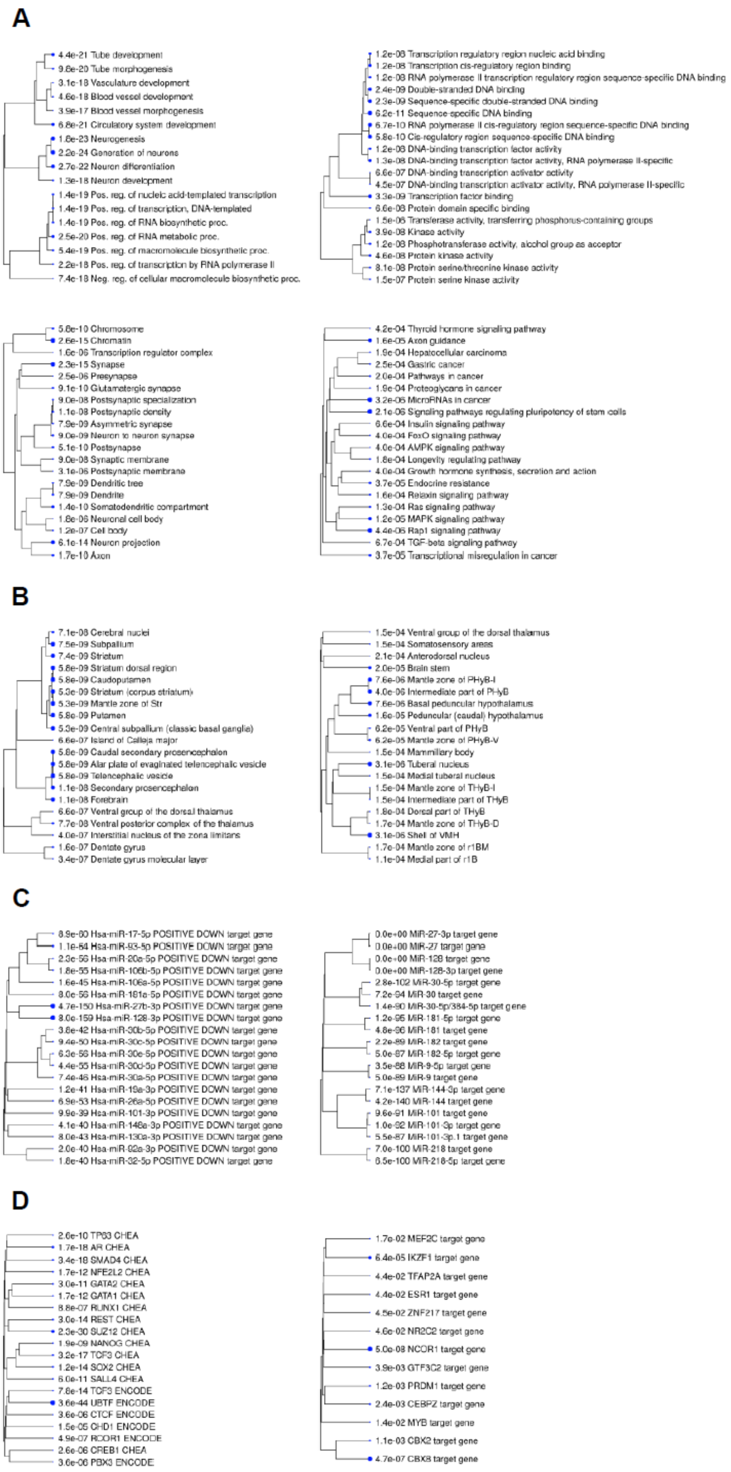
A
4.4e-21 Tube development
9.8e-20 Tube morphogenesis
3.1e-18 Vasculature development
4.6e-18 Blood vessel development
3.9e-17 Blood vessel morphogenesis
6.8e-21 Circulatory system development
1.8e-23 Neurogenesis
2.2e-24 Generation of neurons
2.7e-22 Neuron differentiation
1.3e-18 Neuron development
1.4e-19 Pos. reg. of nucleic acid-templated transcription
1.4e-19 Pos. reg. of transcription, DNA-templated
1.4e-19 Pos. reg. of RNA biosynthetic proc.
2.5e-20 Pos. reg. of RNA metabolic proc.
5.4e-19 Pos. reg. of macromolecule biosynthetic proc.
2.2e-18 Pos. reg. of transcription by RNA polymerase II
7.4e-18 Neg. reg. of cellular macromolecule biosynthetic proc.
1.2e-08 Transcription regulatory region nucleic acid binding
1.2e-08 Transcription cis-regulatory region binding
1.2e-08 RNA polymerase II transcription regulatory region sequence-specific DNA binding
2.4e-09 Double-stranded DNA binding
2.3e-09 Sequence-specific double-stranded DNA binding
6.2e-11 Sequence-specific DNA binding
6.7e-10 RNA polymerase II cis-regulatory region sequence-specific DNA binding
5.8e-10 Cis-regulatory region sequence-specific DNA binding
1.2e-08 DNA-binding transcription factor activity
1.3e-08 DNA-binding transcription factor activity, RNA polymerase II-specific
6.6e-07 DNA-binding transcription activator activity
4.5e-07 DNA-binding transcription activator activity, RNA polymerase II-specific
3.3e-09 Transcription factor binding
6.6e-08 Protein domain specific binding
1.5e-06 Transferase activity, transferring phosphorus-containing groups
3.9e-08 Kinase activity
1.2e-08 Phosphotransferase activity, alcohol group as acceptor
4.6e-08 Protein kinase activity
8.1e-08 Protein serine/threonine kinase activity
1.5e-07 Protein serine kinase activity
5.8e-10 Chromosome
2.6e-15 Chromatin
1.6e-06 Transcription regulator complex
2.3e-15 Synapse
2.5e-06 Presynapse
9.1e-10 Glutamatergic synapse
9.0e-08 Postsynaptic specialization
1.1e-08 Postsynaptic density
7.9e-09 Asymmetric synapse
9.0e-09 Neuron to neuron synapse
5.1e-10 Postsynapse
9.0e-08 Synaptic membrane
3.1e-06 Postsynaptic membrane
7.9e-09 Dendritic tree
7.9e-09 Dendrite
1.4e-10 Somatodendritic compartment
1.8e-06 Neuronal cell body
1.2e-07 Cell body
6.1e-14 Neuron projection
1.7e-10 Axon
4.2e-04 Thyroid hormone signaling pathway
1.6e-05 Axon guidance
1.9e-04 Hepatocellular carcinoma
2.5e-04 Gastric cancer
2.0e-04 Pathways in cancer
1.9e-04 Proteoglycans in cancer
3.2e-06 MicroRNAs in cancer
2.1e-06 Signaling pathways regulating pluripotency of stem cells
6.6e-04 Insulin signaling pathway
4.0e-04 FoxO signaling pathway
4.0e-04 AMPK signaling pathway
1.8e-04 Longevity regulating pathway
4.0e-04 Growth hormone synthesis, secretion and action
3.7e-05 Endocrine resistance
1.6e-04 Relaxin signaling pathway
1.3e-04 Ras signaling pathway
1.2e-05 MAPK signaling pathway
4.4e-06 Rap1 signaling pathway
6.7e-04 TGF-beta signaling pathway
3.7e-05 Transcriptional misregulation in cancer
B
7.1e-08 Cerebral nuclei
7.5e-09 Subpallium
7.4e-09 Striatum
5.8e-09 Striatum dorsal region
5.8e-09 Caudoputamen
5.3e-09 Striatum (corpus striatum)
5.3e-09 Mantle zone of Str
5.8e-09 Putamen
5.3e-09 Central subpallium (classic basal ganglia)
6.6e-07 Island of Calleja major
5.8e-09 Caudal secondary prosencephalon
5.8e-09 Alar plate of evaginated telencephalic vesicle
5.8e-09 Telencephalic vesicle
1.1e-08 Secondary prosencephalon
1.1e-08 Forebrain
6.6e-07 Ventral group of the dorsal thalamus
7.7e-08 Ventral posterior complex of the thalamus
4.0e-07 Interstitial nucleus of the zona limitans
1.6e-07 Dentate gyrus
3.4e-07 Dentate gyrus molecular layer
1.5e-04 Ventral group of the dorsal thalamus
1.5e-04 Somatosensory areas
2.1e-04 Anterodorsal nucleus
2.0e-05 Brain stem
7.6e-06 Mantle zone of PHyB-I
4.0e-06 Intermediate part of PHyB
7.6e-06 Basal peduncular hypothalamus
1.6e-05 Peduncular (caudal) hypothalamus
6.2e-05 Ventral part of PHyB
6.2e-05 Mantle zone of PHyB-V
1.5e-04 Mammillary body
3.1e-06 Tuberal nucleus
1.5e-04 Medial tuberal nucleus
1.5e-04 Mantle zone of THyB-I
1.5e-04 Intermediate part of THyB
1.8e-04 Dorsal part of THyB
1.7e-04 Mantle zone of THyB-D
3.1e-06 Shell of VMH
1.7e-04 Mantle zone of r1BM
1.1e-04 Medial part of r1B
C
8.9e-60 Hsa-miR-17-5p POSITIVE DOWN target gene
1.1e-64 Hsa-miR-93-5p POSITIVE DOWN target gene
2.3e-56 Hsa-miR-20a-5p POSITIVE DOWN target gene
1.8e-55 Hsa-miR-106b-5p POSITIVE DOWN target gene
1.6e-45 Hsa-miR-106a-5p POSITIVE DOWN target gene
8.0e-56 Hsa-miR-181a-5p POSITIVE DOWN target gene
4.7e-150 Hsa-miR-27b-3p POSITIVE DOWN target gene
8.0e-159 Hsa-miR-128-3p POSITIVE DOWN target gene
3.8e-42 Hsa-miR-30b-5p POSITIVE DOWN target gene
9.4e-50 Hsa-miR-30c-5p POSITIVE DOWN target gene
6.3e-56 Hsa-miR-30e-5p POSITIVE DOWN target gene
4.4e-55 Hsa-miR-30d-5p POSITIVE DOWN target gene
7.4e-46 Hsa-miR-30a-5p POSITIVE DOWN target gene
1.2e-41 Hsa-miR-19a-3p POSITIVE DOWN target gene
6.9e-53 Hsa-miR-26a-5p POSITIVE DOWN target gene
9.9e-39 Hsa-miR-101-3p POSITIVE DOWN target gene
4.1e-40 Hsa-miR-146a-3p POSITIVE DOWN target gene
8.0e-43 Hsa-miR-130a-3p POSITIVE DOWN target gene
2.0e-40 Hsa-miR-92a-3p POSITIVE DOWN target gene
1.8e-40 Hsa-miR-32-5p POSITIVE DOWN target gene
0.0e+00 MiR-27-3p target gene
0.0e+00 MiR-27 target gene
0.0e+00 MiR-128 target gene
0.0e+00 MiR-128-3p target gene
2.8e-102 MiR-30-5p target gene
7.2e-94 MiR-30 target gene
1.4e-90 MiR-30-5p/384-5p target gene
1.2e-95 MiR-181-5p target gene
4.8e-96 MiR-181 target gene
2.2e-89 MiR-182 target gene
5.0e-87 MiR-182-5p target gene
3.5e-88 MiR-9-5p target gene
5.0e-89 MiR-9 target gene
7.1e-137 MiR-144-3p target gene
4.2e-140 MiR-144 target gene
9.6e-91 MiR-101 target gene
1.0e-92 MiR-101-3p target gene
5.5e-87 MiR-101-3p.1 target gene
7.0e-100 MiR-218 target gene
6.5e-100 MiR-218-5p target gene
D
2.6e-10 TP63 CHEA
1.7e-18 AR CHEA
3.4e-18 SMAD4 CHEA
1.7e-12 NFE2L2 CHEA
3.0e-11 GATA2 CHEA
1.7e-12 GATA1 CHEA
8.8e-07 RUNX1 CHEA
3.0e-14 REST CHEA
2.3e-30 SUZ12 CHEA
1.9e-09 NANOG CHEA
3.2e-17 TCF3 CHEA
1.2e-14 SOX2 CHEA
6.0e-11 SALL4 CHEA
7.8e-14 TCF3 ENCODE
3.6e-44 UBTF ENCODE
3.6e-06 CTCF ENCODE
1.5e-05 CHD1 ENCODE
4.9e-07 RCOR1 ENCODE
2.6e-06 CREB1 CHEA
3.6e-06 PBX3 ENCODE
1.7e-02 MEF2C target gene
6.4e-05 IKZF1 target gene
4.4e-02 TFAP2A target gene
4.4e-02 ESR1 target gene
4.5e-02 ZNF217 target gene
4.6e-02 NR2C2 target gene
5.0e-08 NCOR1 target gene
3.9e-03 GTF3C2 target gene
1.2e-03 PRDM1 target gene
2.4e-03 CEBPZ target gene
1.4e-02 MYB target gene
1.1e-03 CBX2 target gene
4.7e-07 CBX8 target gene

Figure S2. Functional annotation analysis of the miR-128-3p target genes.
A hierarchical clustering tree of gene ontology pathways (A), sub-brain localization (B), co-targeting (C), and transcription factor network (D) enrichment analysis generated by ShinyGO for the miR-128-3p target genes (n=1225). Larger dots indicate more significant p-values. FDR cutoff >0.05.

A Gene Ontology: Biological Processes, Molecular Function, Cellular Compartment and KEGG pathways [13] (Table S1/Spreadsheet: 7-10).

B Up-regulation (left) and down-regulation (right) of miR-128-3p target genes within sub-brain localizations [19] (Table S1/Spreadsheet: 11-12).

C Targets of miR-128-3p by TargetScan analysis [16] and inverse correlation of miR-128-3p targets by Diana web server analysis [20] (Table S1/Spreadsheet: 13-14).

D Targets of miR-128 co-targeted by transcription factors by Enrichr (gene set enrichment analysis tool) [17] and ENCODE (Encyclopaedia of DNA Elements) portal [21] (Table S1/Spreadsheet: 15-16).

Table S1.

Spreadsheet 1: miEAA - miRNA Enrichment and Annotation – Full analysis results
Spreadsheet 2: dataset pertaining to the Figure S1A – total dataset
Spreadsheet 3: dataset pertaining to the Figure S1A – dataset upon cutoff
Spreadsheet 4: dataset pertaining to the Figure S1B
Spreadsheet 5: dataset pertaining to the Figure S1C
Spreadsheet 6: TargetScan 8.0 – list of hsa-miR-128-3p targets
Spreadsheet 7: dataset pertaining to the Figure S2A - upper left
Spreadsheet 8: dataset pertaining to the Figure S2A - upper right
Spreadsheet 9: dataset pertaining to the Figure S2A - bottom left
Spreadsheet 10: dataset pertaining to the Figure S2A - bottom right
Spreadsheet 11: dataset pertaining to the Figure S2B - left
Spreadsheet 12: dataset pertaining to the Figure S2B - right
Spreadsheet 13: dataset pertaining to the Figure S2C - left
Spreadsheet 14: dataset pertaining to the Figure S2C - right
Spreadsheet 15: dataset pertaining to the Figure S2D - left
Spreadsheet 16: dataset pertaining to the Figure S2D - right

SUPPLEMENTAL REFERENCES

1. Keller, A., et al., *miRNATissueAtlas2: an update to the human miRNA tissue atlas.* Nucleic Acids Res, 2022. **50**(D1): p. D211-D221.
2. Fehlmann, T., E. Meese, and A. Keller, *Exploring ncRNAs in Alzheimer's disease by miRMaster.* Oncotarget, 2017. **8**(3): p. 3771-3772.
3. Fehlmann, T., et al., *Web-based NGS data analysis using miRMaster: a large-scale meta-analysis of human miRNAs.* Nucleic Acids Res, 2017. **45**(15): p. 8731-8744.
4. Fehlmann, T., et al., *miRMaster 2.0: multi-species non-coding RNA sequencing analyses at scale.* Nucleic Acids Res, 2021. **49**(W1): p. W397-W408.
5. Kozomara, A., M. Birgaoanu, and S. Griffiths-Jones, *miRBase: from microRNA sequences to function.* Nucleic Acids Res, 2019. **47**(D1): p. D155-D162.
6. Guttman, M., et al., *Chromatin signature reveals over a thousand highly conserved large non-coding RNAs in mammals.* Nature, 2009. **458**(7235): p. 223-7.
7. Consortium, R.N., *RNAcentral 2021: secondary structure integration, improved sequence search and new member databases.* Nucleic Acids Res, 2021. **49**(D1): p. D212-D220.
8. Chan, P.P. and T.M. Lowe, *GtRNAdb 2.0: an expanded database of transfer RNA genes identified in complete and draft genomes.* Nucleic Acids Res, 2016. **44**(D1): p. D184-9.

9. Fang, S., et al., *NONCODEV5: a comprehensive annotation database for long non-coding RNAs.* Nucleic Acids Res, 2018. **46**(D1): p. D308-D314.
10. Aparicio-Puerta, E., et al., *miEAA 2023: updates, new functional microRNA sets and improved enrichment visualizations.* Nucleic Acids Res, 2023. **51**(W1): p. W319-W325.
11. Ge, S.X., D. Jung, and R. Yao, *ShinyGO: a graphical gene-set enrichment tool for animals and plants.* Bioinformatics, 2020. **36**(8): p. 2628-2629.
12. Jin, Z., et al., *KEGG tools for classification and analysis of viral proteins.* Protein Sci, 2023. **32**(12): p. e4820.
13. Thomas, P.D., et al., *PANTHER: Making genome-scale phylogenetics accessible to all.* Protein Sci, 2022. **31**(1): p. 8-22.
14. Hawrylycz, M.J., et al., *An anatomically comprehensive atlas of the adult human brain transcriptome.* Nature, 2012. **489**(7416): p. 391-399.
15. Paraskevopoulou, M.D., et al., *DIANA-microT web server v5.0: service integration into miRNA functional analysis workflows.* Nucleic Acids Res, 2013. **41**(Web Server issue): p. W169-73.
16. Agarwal, V., et al., *Predicting effective microRNA target sites in mammalian mRNAs.* Elife, 2015. **4**.
17. Kuleshov, M.V., et al., *Enrichr: a comprehensive gene set enrichment analysis web server 2016 update.* Nucleic Acids Res, 2016. **44**(W1): p. W90-7.
18. Consortium, E.P., *An integrated encyclopedia of DNA elements in the human genome.* Nature, 2012. **489**(7414): p. 57-74.
19. Ding, S.L., et al., *Comprehensive cellular-resolution atlas of the adult human brain.* J Comp Neurol, 2016. **524**(16): p. 3127-481.
20. Maragkakis, M., et al., *DIANA-microT Web server upgrade supports Fly and Worm miRNA target prediction and bibliographic miRNA to disease association.* Nucleic Acids Res, 2011. **39**(Web Server issue): p. W145-8.
21. Luo, Y., et al., *New developments on the Encyclopedia of DNA Elements (ENCODE) data portal.* Nucleic Acids Res, 2020. **48**(D1): p. D882-D889.
