# Supplementary material for: MiR-128-3p – a gray eminence of the human central nervous system
Source: Mol Ther Nucleic Acids. 2024 Feb 6;35(1):102141. doi: 10.1016/j.omtn.2024.102141 (PMC10899074; doi:10.1016/j.omtn.2024.102141)
Supplement: Document S2. Article plus supplemental information [file mmc3.pdf]

Molecular Therapy
Nucleic Acids
Review

MiR-128-3p – a gray eminence of the human central nervous system

Klaudia Kiel,[1,3] Sylwia Katarzyna Król,[2,3] Agnieszka Bronisz,[1] and Jakub Godlewski[2]

[1]Tumor Microenvironment Laboratory, Mossakowski Medical Research Institute, Polish Academy of Sciences, 5 Pawińskiego Street, Warsaw, Poland; [2]Department of Neurooncology, Mossakowski Medical Research Institute, Polish Academy of Sciences, 5 Pawińskiego Street, Warsaw, Poland

MicroRNA-128-3p (miR-128-3p) is a versatile molecule with multiple functions in the physiopathology of the human central nervous system. Perturbations of miR-128-3p, which is enriched in the brain, contribute to a plethora of neurodegenerative disorders, brain injuries, and malignancies, as this miRNA is a crucial regulator of gene expression in the brain, playing an essential role in the maintenance and function of cells stemming from neuronal lineage. However, the differential expression of miR-128-3p in pathologies underscores the importance of the balance between its high and low levels. Significantly, numerous reports pointed to miR-128-3p as one of the most depleted in glioblastoma, implying it is a critical player in the disease's pathogenesis and thus may serve as a therapeutic agent for this most aggressive form of brain tumor. In this review, we summarize the current knowledge of the diverse roles of miR-128-3p. We focus on its involvement in the neurogenesis and pathophysiology of malignant and neurodegenerative diseases. We also highlight the promising potential of miR-128-3p as an antitumor agent for the future therapy of human cancers, including glioblastoma, and as the linchpin of brain development and function, potentially leading to the development of new therapies for neurological conditions.

INTRODUCTION

MicroRNAs are small (20–23 nucleotides), frequently highly conserved regulatory non-coding RNAs (ncRNA) that control post-transcriptional regulation of the expression of protein-coding genes.[1] MicroRNAs prevent protein production by interacting with the complementary sequence within the 3′UTR region of target mRNA, destabilizing the transcript or blocking protein synthesis at ribosomes. MicroRNAs can also interact with the 5′UTR region of mRNA or coding sequences, serve as a ligand (e.g., Toll-like receptors), or interact with other ncRNA.[2,3] They are found in body fluids, extracellular vesicles (EV), and high-density lipoproteins, making them a good source of biomarkers for detecting, diagnosing, and prognosis of various cancers.[4–7] In Figure 1 we present the timeline of the most crucial events in the discovery of microRNAs.[8–20]

Overview of miR-128-3p

The development and physiological functionality of a neuronal component of the central nervous system (CNS) requires the coordination of various regulatory systems that implement appropriate timing, networking, and microenvironmental organization toward terminal differentiation. A large volume of evidence implicates microRNAs playing crucial roles in multiple biological functions such as proliferation, differentiation, or apoptosis.[3,21] MiR-128-3p, encoded by two separate genes, *hsa-MIR128-1* and *hsa-MIR128-2* (www.ensembl.org), is one of the most highly expressed microRNAs across the CNS and surroundings tissues (Figure 2).[22]

MiR-128-3P BIOGENESIS

Current classification distinguishes three major microRNA biogenesis pathways: canonical microRNA, canonical intronic microRNA, and non-canonical intronic small RNA (mirtron).[23] The biogenesis of the *hsa-MIR128-1* pathway is the canonical one and is regulated by polymerase II (Pol II), yet both can modulate the *hsa-MIR128-2* pathway: Pol II host gene promotors and Pol III intronic promotors.[24,25] *Hsa-MIR128-1* is embedded within the intron of protein-coding gene *R3HDM1* on chromosome 2 (q21.3), while *hsa-MIR128-2* is located in chromosome 3 (p22.3) within the intron of the protein-coding *ARPP-21* gene[26] (Figure 3). The microRNA gene *R3HDM1/ARPP-21* is transcribed by RNA Pol II/III, resulting in the long primary transcript (pri-microRNA). The typical pri-microRNA has several thousand base pairs and contains a cap structure (7-methyl-guanosine) at the 5′ end, a poly-A tail at the 3′ one, and multiple local hairpin loops.[24] Such pri-microRNA is further cleaved by a microprocessor complex (consisting of endoribonuclease DROSHA and RNA-binding protein DGCR8) in the nucleus, generating precursor microRNA (pre-microRNA), a 60–100-nucleotide stem-loop structure with ∼2-nucleotide 3′ overhang. This motif of pre-microRNA is then recognized by Exportin-5 (XPO5)/Ran-guanosine triphosphate (Ran-GTP) and translocated from the nucleus to the cytoplasm, where it is further processed by the DICER complex to generate a short microRNA duplex. Finally, such a duplex is unwound, and strands are separated. One serves as the mature microRNA, while another is degraded. Crucially, although both ∼80-nucleotide-long pre-miR-128 form different stem-loop structures, they yield identical mature miR-128-3p after processing by DICER.[1,23,26]

https://doi.org/10.1016/j.omtn.2024.102141.

[3]These authors contributed equally

Correspondence: Jakub Godlewski, Department of Neurooncology, Mossakowski Medical Research Institute, Polish Academy of Sciences, 5 Pawińskiego Street, Warsaw, Poland.
E-mail: jgodlewski@imdik.pan.pl

This is an open access article under the CC BY license (http://creativecommons.org/licenses/by/4.0/).

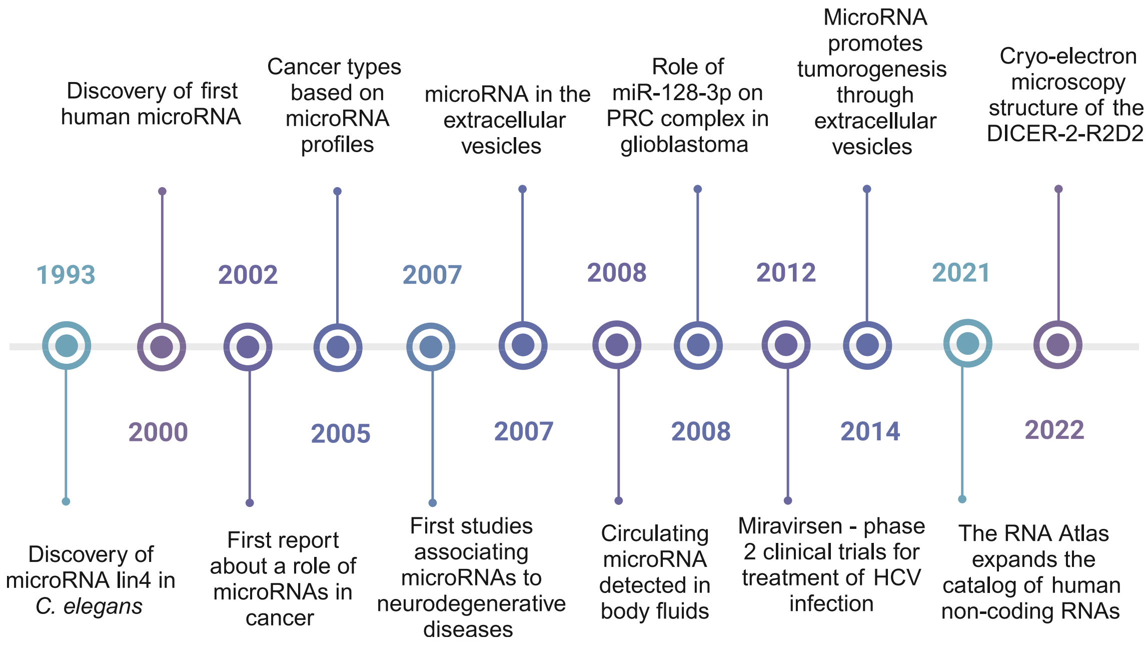

Figure 1. The milestones of microRNA discovery, including miR-128-3p

Both protein-coding host genes are highly and predominantly expressed in the brain, suggesting the mechanism for organ specificity of miR-128's expression. However, while the expression of *R3HDM1* is particularly high in the cerebral cortex, where it overlaps with high levels of miR-128-3p (Figures S1B and S1C), *ARPP21* is strongly expressed in basal ganglia.[27] Notably, both host genes are lowly expressed in glioblastoma, suggesting a common regulatory pathway for the host gene and its embedded microRNA.[28] Few reports so far have focused on distinguishing *MIR128-1* and *MIR128-2* loci. Interestingly, Tan et al. demonstrated that the *mmu-MIR128-2* locus is responsible for generating a majority of mature mmu-miR-128-3p in mice[29]; however, it remains to be investigated whether this finding applies to humans as well, as mechanisms responsible for the expression of non-coding RNAs are generally not well-conserved between species.

As to the mature transcripts, the main detected in the brain tissue in humans and mice is miR-128-3p (target sequence CACUGUGA[30]), whose expression is somewhat associated with miR-128-1-5p (CGGCCCCA). Nevertheless, the expression of miR-128-5p (regardless of locus of origin) is barely detectable, while miR-128-3p is one of the most enriched in the brain; therefore, the networking/targeting analysis presented in this report focuses on miR-128-3p.

FUNCTIONS OF miR-128-3P IN THE PHYSIOPATHOLOGY OF THE HUMAN CNS

MiR-128-3p exhibits strict tissue-specific expression patterns (as depicted in Figures 2 and 4), being prominently observed in differentiating neuronal cells[29,31] and favoring the neocortex over the cerebellum. A report by He et al., which contrasts miR-128-3p expression in specific brain regions, supports this tissue-centric perspective.[32]

MiR-128-3p regulates the expression of many genes, including those encoding for ion channels and transporters, which are critical for maintaining the electrical activity of neurons that contribute to neuronal excitability and neurotransmitter-driven motor activity.[29] Predominantly high expression of miR-128-3p was found in neurons and synapses,[21,22] while its significantly lower level was shown in astrocytes and oligodendrocytes.[29]

On a different note, miR-128-3p actively participates in embryonic brain development by regulating key genes, including *PCM-1* (Pericentriolar material 1) and *PHF6* (PHD finger protein 6) in neuronal progenitor cells. This regulation promoted neurogenesis while inhibiting proliferation in the developing neocortex, while removal of miR-128-3p enhanced cell division, reducing neuron formation.[33]

The intricacies of miR-128's role extend beyond broadly categorized developmental stages. In the mammalian neocortex, where the generation of different neuronal types is precisely timed, miR-128's expression dynamically changes during neurogenesis, when it influences stem cell competence, timing neocortical layer formation, and specifying laminar fates. As neurogenesis progresses, miR-128-3p expression decreases in stem cells while maintaining specific differences in microRNA expression in neurons. MiR-128-3p, along with other microRNAs such as miR-9 and let-7, collectively modulate stem cell competence in a neurogenic-stage-specific manner, influencing the timing and fate of neuron production.[34]

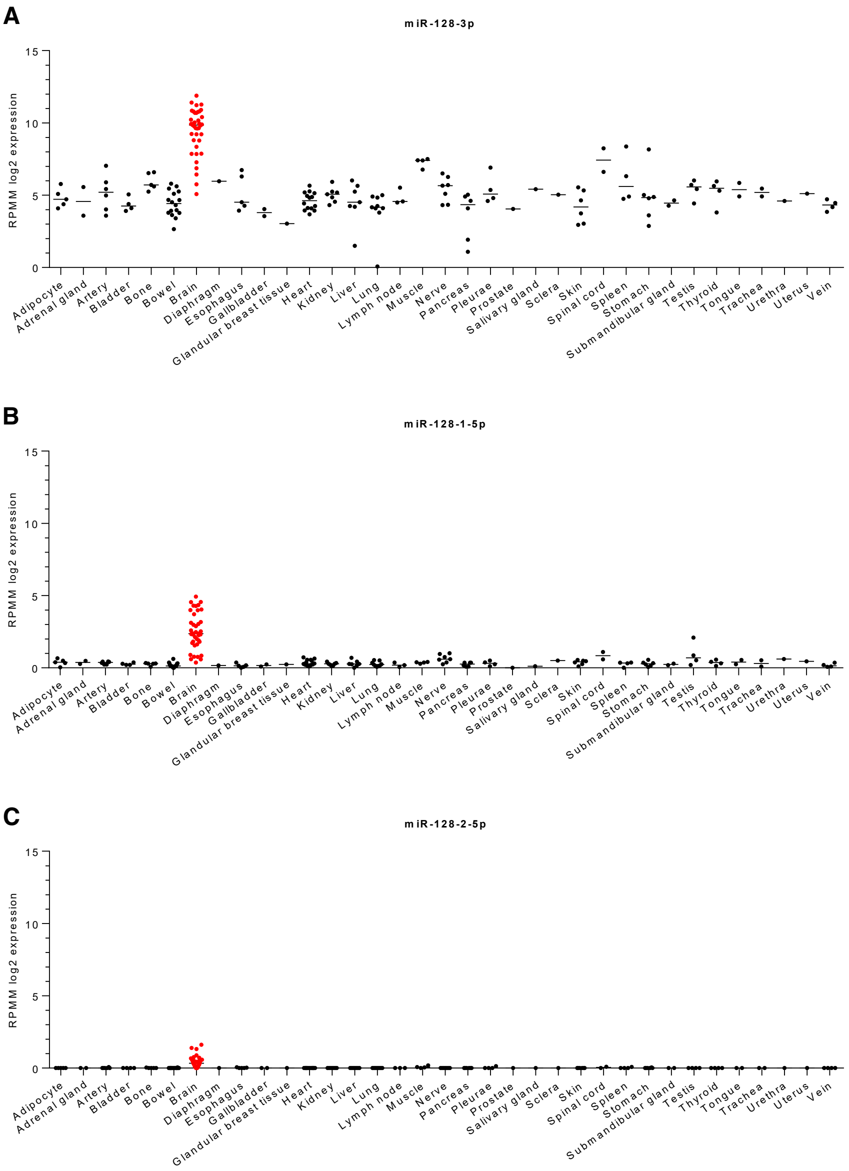

Figure 2. The expression of mature miR-128-3p, miR-128-1-5p, and miR-128-2-5p across normal human tissues and organs

Scatter plots of miR-128-3p (A), miR-128-1-5p (B), and miR-128-2-5p (C) expression (shown as log2 RPMM; reads per million mapped reads) in human tissues and organs. The scatterplots generated by GraphPad Prism (ver. 10.0) using the datasets obtained from Human microRNA Tissue Atlas (https://ccb-web.cs.uni-saarland.de/tissueatlas2), accessed on December 2023). The number of specimens: adipocyte n = 5, adrenal gland n = 2, artery n = 6, bladder n = 4, bone n = 5, bowel n = 16, brain n = 39, diaphragm n = 1, esophagus n = 5, gallbladder n = 2, glandular breast tissue n = 1, heart n = 14, kidney n = 7, liver n = 7, lung n = 9, lymph node n = 3, muscle n = 4, nerve n = 7, pancreas n = 6, pleurae n = 4, prostate n = 1, salivary gland n = 1, sclera n = 1, skin n = 6, spinal cord n = 2, spleen n = 4, stomach n = 6, submandibular gland n = 2, testis n = 4, thyroid n = 4, tongue n = 2, trachea n = 2, urethra n = 1, uterus n = 1, vein n = 4.

In summary, miR-128-3p plays a crucial role in mammalian brain development, both exhibiting strict tissue-specific expression patterns and overseeing developmental stage trajectory. Its involvement spans neural progenitor cell regulation to orchestrating the formation of complex neuronal networks during brain circuit development. To the point, disruptions in miR-128-3p homeostasis are associated with neurodevelopmental disorders, emphasizing its potential as a therapeutic target for conditions like autism spectrum disorder, schizophrenia, and epilepsy.[35–39]

MiR-128-3p exhibits cell-type-specific expression in brain tissue. It is strongly expressed in neurons and neural progenitors[34] but weakly expressed in mature astrocytes, and its levels are even lower in glioblastoma cells,[40] suggesting an active suppression mechanism. Although glioblastoma belongs to astrocytomas, and therefore, the prevailing agreement is that astrocytes are cells-of-origin, there are multiple lines of evidence that it originates from neural stem cells (NSCs), NSC-derived glial progenitors, and oligodendrocyte precursor cells.[41–44] The miR-128-3p depletion in glioblastoma tissue and cells is most likely caused by the disrupted activity of microRNA processing machinery[45] but likely involves a combination of many other factors like additional mutations or epigenetic changes in genes involved in microRNA processing or aberrant transcriptional regulation. Other RNA molecules (e.g., circular RNAs[46]) might also compete with miR-128-3p for binding to common mRNA targets or "sponge" it out from the cells, effectively neutralizing its effect., and further research is needed to elucidate the intricate regulatory networks involved. The deregulation of miR-128-3p expression in a cell-specific manner is associated with neurological disorders due to its function in different cell types.[47–50]

MiR-128-3P IN NEURODEGENERATIVE DISEASES, BRAIN DAMAGE, AND INJURIES

Epilepsy

Animal model studies showed that miR-128-3p regulates motor behavior by modulating neuronal signaling networks and excitability. Conditional deletion of both miR-128-3p forms in postnatal neurons in mice resulted in fatal epilepsy. Reintroducing miR-128-3p could thus potentially be an experimental treatment for epilepsy and movement disorders.[29,51] Notably, low levels of miR-128-3p in low-grade gliomas have also been implicated in glioma-associated seizures.[52]

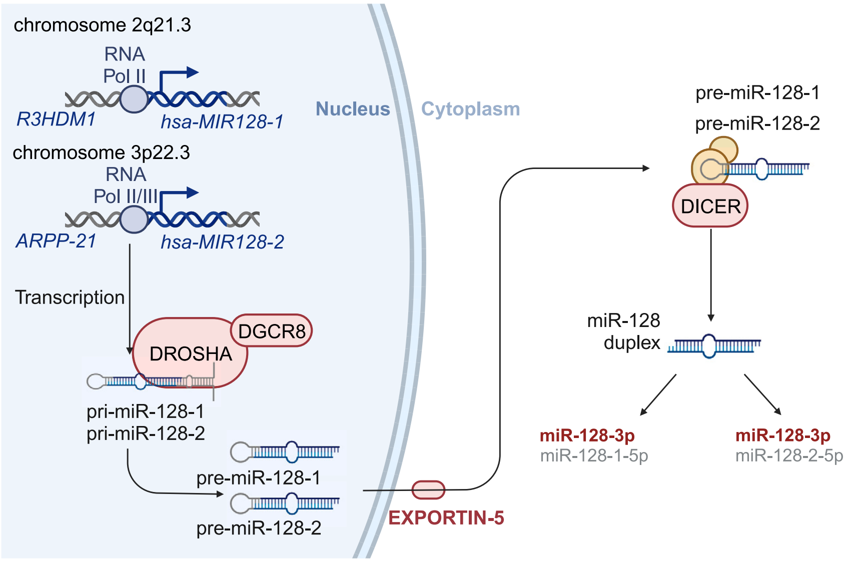

Figure 3. The canonical pathway of miR-128 biogenesis

The *hsa-MIR128-1* gene resides within an intron of the *R3HDM1* protein-coding gene on chromosome 2 (q21.3), whereas the *hsa-MIR128-2* gene is located on chromosome 3 (p22.3) within an intron of the *ARPP-21* protein-coding gene. These genomic loci undergo RNA Pol II/III transcription, yielding an extended primary transcript (pri-microRNA). Subsequently, the microRNA complex, composed of the endoribonuclease DROSHA and the RNA-binding protein DGCR8, cleaves these pri-microRNAs in the nucleus, generating precursor microRNAs (pre-microRNAs). The exportin-5 (XPO5)/Ran-guanosine triphosphate (Ran-GTP) complex recognizes the pre-microRNA, facilitating its translocation from the nucleus to the cytoplasm. Once in the cytoplasm, the DICER complex further processes the pre-microRNA, producing a short microRNA duplex. The duplex undergoes unwinding, with one strand becoming the mature microRNA and the other being degraded. It is important to note that the *hsa-MIR128* gene produces two mature microRNA variants, miR-128-3p, and miR-128-5p, resulting from this processing pathway. Notably, both loci ultimately yield an identical mature miR-128-3p upon DICER processing.

Alzheimer's disease

MiR-128-3p is abnormally enriched in the hippocampal region of both fetal brain and adult patients with Alzheimer's disease (AD),[12,53] while the expression of its direct target peroxisome proliferator activated receptor gamma (PPARG), which reduces amyloid beta protein levels, is down-regulated. Knockout of miR-128-3p unblocks the expression of PPARG and consequently reduces amyloid beta-mediated cytotoxicity and inflammatory response through the inactivation of NF-κB *in vitro*.[54]

Spinal cord injury and neuropathic pain

Spinal cord injury (SCI) leads to permanent motor deficits accompanied by inflammation and oxidative stress, thus resulting in neuronal cell death.[55,56] Overexpression of miR-128-3p has inhibited apoptosis of neurons, inflammation, and improved motor function following SCI via down-regulation of serine/threonine-protein kinase ULK1 and elevation of Fas ligand (FasL).[57] The level of miR-128-3p was significantly decreased in murine microglial cells upon experimentally induced neuropathic pain (NPP) following SCI. The studies showed that overexpression of miR-128-3p improved viability and activation of anti-inflammatory microglial M2 phenotype via down-regulation of the microglial M1 markers (CD86 and CD32) and up-regulation of the M2 phenotypic markers (arginase 1 and CD206).[58] MiR-128-3p also diminished the level/activity of such factors as tumor necrosis factor (TNF-α), interleukin-1β, interleukin-6, p38 mitogen-activated protein kinase (MAPK), or zinc finger E-box binding homeobox 1 (ZEB1) (in microglia) that would potentially constitute novel therapeutic avenues to restrain NPP.[59]

Parkinson's disease

Parkinson's disease (PD) is a neurodegenerative disorder resulting in severe disability due to progressive degeneration of the nigrostriatal dopaminergic pathway.[60,61] The overexpression of miR-128-3p resulted in prominently reduced apoptosis of dopaminergic neurons, contributing to the de-repression of the Wnt/beta catenin signaling pathway protecting neurons against misfolded protein-mediated disorders, such as PD. Consequently, overexpressed miR-128-3p may present a new, potential target for treatment in PD patients.[62,63]

Acute ischemic stroke

Acute ischemic stroke (AIS) occurs when a blockade of blood flow through a brain artery results in a corresponding loss of neurological function.[64,65] Interestingly, the elevated level of miR-128-3p was detected in circulating lymphocytes, neutrophils, and plasma of patients with AIS compared with healthy individuals. It suggests the involvement of miR-128-3p in the process, as it positively correlates with stroke severity. Moreover, the antagomir-mediated depletion of miR-128-2 promoted neuronal cell cycle re-entry and exacerbated ischemia reperfusion-induced neuronal injury.[66]

Multiple sclerosis

Multiple sclerosis (MS) is the most common non-traumatic, autoimmune neurological disorder that disproportionately affects young adults.[67,68] MiR-128-3p is among the most significantly up-regulated microRNA in MS patients when compared with healthy individuals. Its expression was elevated in immune cells, especially naive $CD4^+$ T lymphocytes (T cells). MiR-128-3p was also involved in inhibiting Th2 development and promoting the differentiation of pro-inflammatory Th1 responses in patients with MS, demonstrating a novel function of miR-128-3p in regulating immune cell function, particularly T cells.[69,70]

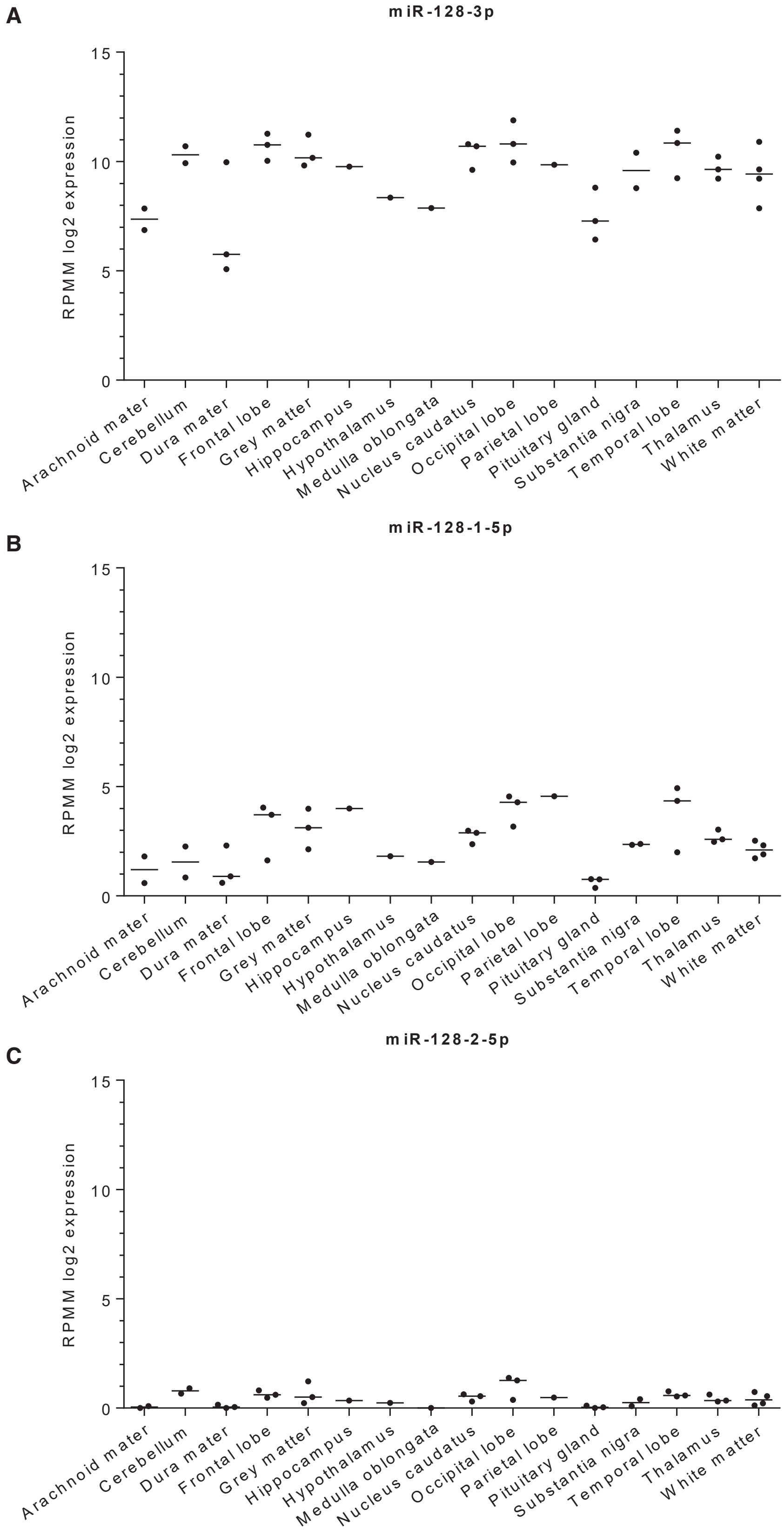

Figure 4. The expression of mature miR-128-3p, miR-128-1-5p, and miR-128-2-5p in brain structures from healthy donors

Scatter plots of miR-128-3p (A), miR-128-1-5p (B), and miR-128-2-5p (C) expression (shown as log2 RPMM; reads per million mapped reads) in human brain structures generated by GraphPad Prism (ver. 10.0) using the datasets obtained from Human microRNA Tissue Atlas (https://ccb-web.cs.uni-saarland.de/tissueatlas2, accessed on December 2023). The number of specimens: arachnoid mater n = 2, cerebellum n = 2, dura mater n = 3, frontal lobe n = 3, gray matter n = 3, hippocampus n = 1, hypothalamus n = 1, medulla oblongata n = 1, nucleus caudatus n = 3, occipital lobe n = 3, parietal lobe n = 1, pituitary gland n = 3, substantia nigra n = 2, temporal lobe n = 3, thalamus n = 3, white matter n = 4.

Table 1. Summary of miR-128-3p deregulations and their effects on neurodegeneration, stroke, and injury of the human CNS

Disease	miR-128-3p deregulation	Effects of deregulation
Alzheimer's disease	Up-regulated	Indirect effect in amyloid-β protein generation
Multiple sclerosis	Up-regulated	Regulation of immune cell functions, particularly T cells
Parkinson's disease	Up-regulated	Protects DA neurons from apoptosis
Acute ischemic stroke	Up-regulated	Regulation of immune cell functions
Epilepsy	Down-regulated	Regulation of motor behavior
Spinal cord injury and neuropathic pain	Down-regulated	Involved in neuronal programmed cell death

Summary of miR-128-3p in neurodegenerative diseases and brain damage

Abundantly expressed in the human brain (primarily in neurons), miR-128-3p plays a crucial role in developing CNS and maintaining its physiological functions. MiR-128-3p controls critical steps in committing toward neuronal lineage and maturing into terminally differentiated neurons, inhibiting cell migration or proliferation. The inverse correlation of miR-128-3p expression levels in non-malignant CNS diseases emphasizes the importance of its balanced expression, suggesting the potential of opposite therapeutic applications (summarized in Table 1).

MiR-128-3P IN MALIGNANCIES OF THE CNS

The expression of miR-128-3p in brain tumors is strongly inversely correlated to the grade of malignancy. The lowest expression of miR-128-3p was found in glioblastomas (grade 4).[71,72] In contrast, relatively high levels of miR-128-3p were apparent in oligodendrogliomas (grade 2 or 3).[71,73] No significant differences were detected in the expression of miR-128-3p between the healthy cohorts and the patients with meningiomas (grade 1–3) or pituitary tumors,[71,72,74] while it was significantly suppressed in more aggressive pituitary adenomas.[74] Thus, as the miR-128-3p expression in benign and low-grade brain malignancies is not much different from expression in non-malignant cells, miR-128-3p was not scrutinized in these pathologies. Instead, a broader spectrum of miR-128-3p deregulations was carefully investigated in astrocytomas (grades 2–4),[75,76] and glioblastoma, where a reduced level of miR-128-3p was correlated with worse patient outcomes.[77–79]

Medulloblastoma

Medulloblastoma grade 3 or 4[80] is the most common malignant brain tumor in children, accounting for nearly 10% of all pediatric brain tumors.[81] In this tumor type, miR-128-3p is severely suppressed compared with the normal cerebellum. Reintroducing miR-128-3p into medulloblastoma cells inhibited their growth *in vitro* by increasing the level of reactive oxygen species (ROS) and promoting cellular senescence through targeting a proto-oncogene *BMI1* (B cell-specific Moloney murine leukemia virus integration site 1).[82]

Pituitary adenoma

Most pituitary tumors are benign, but more aggressive cases also exist. The mechanisms responsible for their genesis and progression into more aggressive forms remain unknown.[83–85] In the aggressive pituitary adenoma type, miR-128-3p is one of the few microRNAs suppressed compared with control tissues. Its reintroduction into pituitary cancer cells inhibits their colony-forming ability and invasiveness *in vitro*. Furthermore, as in medulloblastoma, it interacts with its target *BMI1*. *BMI1* affected *PTEN* (phosphatase and tensin homolog) expression levels and *AKT1* (AKT serine/threonine kinase 1) activity by binding the *PTEN* promoter in pituitary tumor cells.[74]

Glioblastoma

Glioblastoma, a grade 4 malignancy,[80] is the most frequent and aggressive brain tumor in adults, accounting for more than 80% of primary (non-metastatic) brain tumors.[81] It is characterized by rapid cell proliferation and infiltrative growth, thus spreading robustly into surrounding healthy brain tissue. Glioblastoma's high intratumoral genetic and molecular heterogeneity also makes it one of the most difficult human malignancies to combat. The co-existence of several cell sub-populations enhances the heterogeneity with diverse genetic, transcriptional, and functional backgrounds within the bulk tumor.[86–89] Prominent among these sub-populations are glioblastoma stem-like cells (GSCs), a subset of undifferentiated cells capable of self-renewal and multi-lineage differentiation. They are considered tumor-initiating cells that render cancer resistant to conventional anti-glioblastoma therapies,[90–93] leading to inevitable recurrence and fatal outcomes.[94,95] High-throughput genomic, epigenomic, and transcriptomic profiling of glioblastoma cells revealed three main subtypes harboring distinct molecular and phenotypic landscapes that affect response to the therapy and clinical outcome: mesenchymal, proneural, and classical subtypes.[96–100] Currently, available treatment options for glioblastoma, including maximally safe surgical resection, radiation, and adjuvant chemotherapy, are ineffective, leading to only incrementally improved survival of the patients. Despite the significant recent advances in diagnostics and development of therapeutic modalities, coupled with growing knowledge of this malignant brain tumor's genetic and molecular aspects, the median survival is only 14–16 months.[101,102] Some recent reports from high-throughput sequencing strategies showed a response to treatment-driven clonal evolution of glioblastoma cells manifested in different transcriptional signatures at primary diagnosis and relapse. These genetic and molecular shifts are associated with the inter-subclass transition upon therapy with temozolomide[103,104] and radiation.[104] Evidence suggests some novel genetic, epigenetic, and molecular targets for potentially treating malignant brain tumors, including glioblastoma.[93,105–107] Among them, ncRNAs (e.g., microRNA) seem the most emerging due to their widespread occurrence in the human genome (only about 1.5% of total RNA is translated into proteins) and crucial roles in regulating gene expression.[108] Therefore, ncRNAs, including miR-128-3p, and their dysregulations are

Table 2. Summary of miR-128-3p deregulations and their effects on the malignancies of the human CNS

Disease	miR-128-3p deregulation	Effects of deregulation
Medulloblastoma	Down-regulated	Cancer cells growth
Pituitary adenoma	Down-regulated	Colony-forming ability and invasiveness
Glioblastoma	Down-regulated	GSC viability and proliferation, GSC self-renewal and radio-resistance

considered promising targets for novel epigenetic-based therapies for glioblastoma.[109–111] Several findings from a large-scale screening of non-coding transcriptome of glioblastoma cells revealed miR-128-3p as one of the most prominently suppressed microRNAs compared with healthy brain tissue.[22,32,71,73,112]

Therefore, miR-128-3p gained remarkable interest, and its functions have been thoroughly studied in brain tumors in recent years. Notably, the level of miR-128-3p was significantly reduced in the brain tissue of young, pre-symptomatic mice genetically modified to develop glioblastomas but not in the brain of wild-type mice, demonstrating that the miR-128-3p suppression is an early event during gliomagenesis.[77] Crucially, the levels of miR-128-3p forms can also differ depending on the genetic and molecular background, as they correlated with the glioblastoma subtypes, with the most reduced expression in the most aggressive mesenchymal one.[96,113]

MiR-128-3p and the regulation of polycomb repressive complexes in glioblastoma

The large-scale profiling of human glioblastoma cells, including whole genome analysis, provided a deep insight into epigenetic mechanisms involved in maintaining and modifying chromatin structure.[114] Epigenetic modifications regulate the state of chromatin and, consequently, control gene expression in normal and tumor cells via suppression or activation of transcription.[115,116] The deregulation of the epigenetic landscape due to dysfunctions in the expression and/or activity of several epigenetic pathways is prominent and well-established in glioblastoma.[117–119] MiR-128-3p was found to be a potent regulator of the expression and activity of proteins forming polycomb repressive complexes (PRCs). PRCs (comprising PRC1 and 2) are remodeling complexes involved in the epigenetic suppression of target genes through chromatin ubiquitination and methylation. PRCs are crucial in maintaining embryonic stem cells, differentiation, and development. Aberrations in PRCs' functionality were linked to several human cancers,[120,121] significantly contributing to the self-renewal/differentiation balance of cancer stem cells,[122–124] including GSCs.[125] Several studies showed that up-regulation of miR-128-3p profoundly reduced the expression of core protein components of PRC1 and PRC2: BMI-1 (Polycomb complex protein BMI-1)[77,126,127] and SUZ12 (Polycomb protein SUZ12),[77,128,129] respectively. The direct targeting of both PRCs by miR-128-3p leads to chromatin re-arrangements, followed by the reprogramming of PRC-dependent genes involved in the enforcement of the stemness program and inter-subclasses transition of brain tumor cells. Functional studies demonstrated a shift of GSCs from the most aggressive mesenchymal subclass toward the proneural one upon miR-128-3p replacement.[130] Reduced viability and neurosphere formation *in vitro,* decreased tumor burden and improved survival in the mouse intracranial xenograft model were observed due to this inter-subtype transition.[130] At the molecular level, the reintroduction of miR-128-3p into GSCs inhibited the activity of both PRC complexes, thus preventing their partial redundancy.[131–133] As the functional consequences of miR-128-3p,[77,126] re-establishment and repression of PRCs subunits, pronounced alterations in histone modifications,[77] as well as loss of stem-like features by GSCs, were observed *in vitro* and *in vivo,* as clearly shown by down-regulation of CD133 (Prominin-1) expression.[77,126] What is more, PRCs are essential players in DNA damage repair via the activation of damage response machinery proteins.[134–136] Thus, the activation of PRCs, evident from increased expression of BMI1 and SUZ12,[77] contributed to the radio-resistance of tumor cells,[137,138] including glioblastoma.[139,140] The reintroduction of miR-128-3p[77,126] significantly inhibited the radiation-induced expression of PRC subunits and consequently enhanced the radiosensitivity of GSCs accompanied by substantial DNA damage.[77,126] Conversely, up-regulation of PRC components and PRC-dependent chromatin modifications that increased clonogenic potential were observed in both mouse and human non-malignant NSCs upon miR-128-3p knockdown.[77]

Summary of miR-128-3p in malignancies of the CNS

In contrast to the neurodegeneration processes we described above, miR-128-3p was significantly down-regulated, and its overexpression was not observed in human malignant tumors of the CNS (summarized in Table 2), possibly due to different targeted genes, depending on the context of disorders and cell/tissue types. Such a low level of miR-128-3p and a significant correlation of its expression with the tumor grade (the lower the expression of miR-128, the more aggressive the tumor) prove the importance of its loss in the pathogenesis of CNS malignancies, specifically glioblastoma.

THE NETWORKING OF miR-128-3P

mRNA targets

The role of miR-128-3p is multifaceted, influencing both physiological and pathological aspects of CNS biology. Its target genes encompass a spectrum of functions, contributing to the complexity of its therapeutic application. Regarding proliferation and differentiation, miR-128-3p targets genes like *RELN* (Reelin)[141] and *PHF6*,[37] impacting neuronal migration, layer formation, and neurite outgrowth. Additionally, miR-128-3p targets synaptic plasticity and memory-related genes, such as *CREB1* (CAMP Responsive element binding protein 1) or *NRXN1* (Neurexin 1),[142] and genes encoding subunits of ion channels and transporters like the Big Potassium (BK) channel.[29] It also regulates neuroinflammatory genes like *HMGB1* (High-mobility group box-1),[143] potentially offering anti-inflammatory and neuroprotective effects.

Similarly, the multitude of putative and experimentally confirmed target mRNAs include both tumor-suppressive and pro-oncogenic genes, rendering the ultimate effect uncertain. For example, miR-128-3p was shown to downregulate tumor suppressor PTEN,[74] which can promote cell survival, thus resulting in uncontrolled cell growth. Conversely, miR-128-3p targets numerous factors that maintain the "stemness" of cancer cells, including epigenetic regulators and master kinases that can act genome-wide and transcriptome-wide, thus curbing their tumorigenicity.[144] Thus, the therapeutic usage of miR-128-3p is not without controversy, as it exhibits divergent effects depending on specific contexts and target co-expression genes and thus can be regarded as a "double-edged sword" in CNS disease therapy.

To grasp the complexity of miR-128-3p′s role in CNS disease therapy, we provided a detailed analysis of its target genes and their interactions, which include a comprehensive list of identified target genes and network analysis of these targets in the brain, showing unequivocal engagement of miR-128-3p in the neuronal tissue-specific gene expression, thus underscoring its crucial role in regulating neuronal development, maintenance, and function (Table S1). Significantly, a large proportion of microRNAs' target genes are transcriptional regulators binding to DNA in a sequence-specific manner, thus enforcing the neuronal tissue-specific ripple effect. The involvement of miR-128-3p in molecular pathways regulating stem cell pluripotency that relies on precise regulatory mechanisms adds another layer of complexity. miR-128-3p participates in these mechanisms by influencing the expression of genes involved in a delicate balance between self-renewal and differentiation. The deregulation of the microRNAome is a common theme in cancer initiation and progression. Interestingly, among targets of the same miR-128-3p that play a role in maintaining neuronal tissue-specific gene expression are also those that drive cancer-specific microRNAome signature in brain tumors[45] (Figure S2A).

MicroRNA-dependent regulation requires their spatiotemporal co-occurrence with target mRNAs. Accordingly, in the healthy brain, miR-128-3p target genes are particularly abundant in various parts of the cortex, where they overlap with high levels of the microRNA; conversely, miR-128-3p target genes are lowly expressed in the brain stem, coinciding with a lower presence of the microRNA (Figures S1B, S1C, and S2B). These correlations suggest tight microRNA surveillance of the transition of target mRNA into a protein product.

MicroRNAs form networks not only with their direct mRNA targets but also with other microRNAs and transcription regulators. For instance, due to the same seed sequence, miR-128-3p and miR-27-3p target almost identical sets of genes (Figure S2C), but both microRNAs' expression in the brain are inversely correlated (Figures S1A and S1B; Table S1), suggesting a coordinated compensatory co-targeting strategy. Such a strategy relies on the target gene's transcription in the brain's anatomic niche-specific manner. In contrast, miR-128-3p and miR-124-3p, whose expression is highly correlated in the brain, do not overlap in terms of their target genes (Figure S2C), so already perceived regulation of neuronal tissue physiology and pathology by these two microRNAs[145] seems more like a site cooperation strategy.

It is also noteworthy that microRNAs can interact with other classes of non-coding RNAs as competing endogenous molecules.[146,147] Decoding miR-128-3p interaction networks with these molecules from large-scale CLIP-Seq data[148] revealed 11 interacting small RNAs, 33 long non-coding RNAs, and 1,035 circular RNAs; however, most of these interactions have no known functional consequences. Among experimentally validated relations, several long-non-coding RNAs were shown to act as either action-enhancing co-partners or inhibitory "sponges," affecting miR-128-3p activity in the pathophysiology of the CNS.[149–151] Similarly, several circular RNAs have been confirmed to enclose miR-128-3p binding elements, which have functional consequences on neuronal tissue homeostasis.[152,153]

Transcription regulators that govern miR-128-3p target genes regulate cell stemness/differentiation status, e.g., *NANOG* (Nanog homeobox), *SOX2* (SRY-box transcription factor 2), *SUZ12,* and neuronal identity, e.g., *REST* (RE1 silencing transcription factor), *RCOR1* (REST corepressor 1). On the other hand, transcription factors that are targets of miR-128-3p are often de-regulated in various malignancies (Figure S2D). These data indicate that miR-128-3p acts hand-in-hand with some transcriptional regulators while controlling some of them directly, suggesting a multilayered network of co-dependencies, most often in the context of neuronal tissues.

The potential for off-target effects adds complexity to miR-128-3p therapeutic use (as is the case for any other microRNA), as microRNA-mediated targeting of a specific gene may unintentionally affect other players with adverse, unintended consequences. Thus, combining miR-128-based therapies with other treatments becomes crucial to mitigate potentially detrimental effects in cell-specific contexts, as we demonstrated previously.[145] Understanding the intricate and context-dependent nature of miR-128-3p's effects on the CNS is paramount for its therapeutic application, requiring careful consideration of particular diseases and target genes. Further research is imperative to unravel the precise mechanisms and consequences of miR-128-3p regulation in different CNS pathologies.

MicroRNAs

MicroRNAs often work cooperatively to regulate overlapping pathways and processes in the brain. While some information is available, it is important to note that microRNA-microRNA interactions are complex, poorly comprehended, and context-dependent; thus, specific mechanisms may vary in different physiological and pathological contexts. However, some potential mechanisms have been proposed. These include competitive binding (microRNAs compete for binding to the same mRNA targets, leading to differential regulation of gene expression[154]), cooperative binding (microRNAs form complexes and jointly bind to mRNA targets, leading to enhanced or synergistic regulatory effects[155]), and indirect interactions (microRNAs indirectly influence each other's expression through affecting signaling

pathways and other regulatory molecules[156]). Understanding these intricate microRNA interactions is crucial for deciphering the complex regulatory mechanisms underlying brain development and disease.

The cooperative strategies of miR-128-3p, with two other microRNAs, miR-124-3p and miR-137, unfold during neurogenesis, where the orchestration of gene expression is paramount.[6,157] Comprehensive genomic analyses revealed that these microRNAs' target gene sets significantly overlap.[158] This synergy is particularly evident in regulating the SP1 (Transcription factor Sp1), forming a highly interconnected network crucial for neural differentiation and thus revealing the molecular interplay during neurogenesis. In gliomagenesis, the loss of miR-124-3p, miR-128-3p, and miR-137[159] creates an environment conducive to aberrant transcriptional regulation. Targeting oncogenic chromatin repressors such as EZH2 (Enhancer of zeste 2 polycomb repressive complex 2 subunit), BMI-1, and SUZ12, the cooperative strategy of these microRNAs disrupts critical survival mechanisms in glioblastoma cells and unveils promising therapeutic avenues. Synchronizing the expression of these microRNAs, as demonstrated in our recent gene therapy approaches, results in significant anticancer synergism in glioblastoma models.[145] What is more, a correlation of expression between genes and microRNAs (according to The Cancer Genome Atlas glioblastoma dataset) revealed a correlation signature of ∼1700 genes that were either positively or negatively associated with microRNAs and stratified the samples into two clusters with the power of outcome prediction (better outcomes: miR-128-3p, miR-124-3p, and miR-1; worse outcomes miR-10b-5p, miR-31-5p, and miR-21-5p).[45]

The therapeutic potential leveraging the cooperative actions of microRNAs to intervene in neuropathological conditions often acts via the PRC2 that drives neuronal differentiation. In this network, besides miR-128-3p, miR-9 and miR-124-3p target and repress the expression of *USP14* (Ubiquitin specific peptidase 14) whose product interacts with and stabilizes EZH2, thereby allowing the repression of EZH2 and the consequent destabilization of REST.[160] Polycomb-like(PCL) proteins and SUZ12, crucial components of PRC2, are targets of miR-34a,[161] a brain tumor suppressor[162] that promotes healthy brain aging, a pathway whose malfunction is a prominent risk factor for neurodegenerative disease.[161] Such regulatory partnership between miR-34a and miR-128-3p[163] suggests a coordinated epigenetic regulatory mechanism that holds sway over the brain microRNAome. Similarly, miR-9, involved in neural stem cell maintenance and differentiation, is capable of interplay with miR-128-3p in regulating the activity of the PRC1 by targeting its component CBX7 (Chromobox protein homolog 7).[164] Simultaneous down-regulation of miR-9 and miR-128-3p leads to the activation of cell cycle regulators Cyclin E and CDK2 by inhibiting their target genes *CDKN2A* (Cyclin-dependent kinase inhibitor 2A) and *CDKN1B* (Cyclin-dependent kinase inhibitor 1B) via the miR-9/CBX7 and miR-128-3p/SUZ12 feedback loop respectively.[165]

Long non-coding RNAs

Increasingly, new evidence shows the direct interplay between long non-coding RNAs (lncRNAs) and microRNAs. LncRNAs function as competing endogenous RNA (ceRNA) of specific microRNAs and, therefore, modulate the expression of downstream target mRNAs.[166] Such "sponging" of microRNAs by lncRNAs suppresses their activity and, therefore, retains the expression of genes targeted by these microRNAs.[167] MiR-128-3p was identified as a target of several lncRNAs, and their direct interactions regulated neurogenesis and affected both CNS and tumor cells. Recent reports demonstrated the vital function of lncRNA PVT1 (Plasmacytoma variant translocation 1)[168] and its regulatory network with miR-128-3p in the pathogenesis of human gliomas. This lncRNA acts as a molecular sponge of miR-128, including miR-128-3p[169] and miR-128-1-5p[170] via specific competitive binding. Therefore, its down-regulation correlated with an increased level of miR-128. Consequently, the burden of glioma was lessened through suppression of growth, proliferation, and migratory/invasive potential.[169,170] Similarly, the binding sites for lncRNA LINC00346 within miR-128-3p have also been identified in glioma cells, and this regulatory network has enhanced tumor progression.[166] More recent findings demonstrated a decreased level of miR-128-3p through a sponge mechanism by lncRNA HCP5 (Histocompatibility leukocyte antigen complex P5). This direct interaction between microRNA and lncRNA regulated the response of glioma cells to ionizing radiation by interfering with cellular senescence as the knockdown of lncRNA HCP5 inhibited cell proliferation and enhanced radiosensitivity in gliomas.[171] The interaction between lncRNA NEAT1 (Nuclear paraspeckle assembly transcript 1) and miR-128-3p that resulted in the sponging of this microRNA contributed not only to glioma pathogenesis,[172] but also to the progression of spinal cord injury-mediated NPP.[173] The sponging of miR-128-3p by lncRNA Peg13 (Paternally expressed 13) resulted in a maintained expression of *SOX13* (SRY-box transcription factor 13) and significantly reduced anesthetic-related neurotoxicity for NSCs in mice.[174] Moreover, competitive interactions between miR-128-3p and lncRNA GAS5 (Growth arrest-specific 5) were involved in hypoxic-ischemic brain damage, as shown in the neonatal rat model.[175]

It thus becomes part of the consensus in the field that lncRNAs regulate the activity of many microRNAs, and the interplay between these types of ncRNAs is considered an emerging regulatory network essential for transcriptome regulation in physiological and pathological conditions. As miR-128-3p seems to be one of the key players among numerous ncRNAs in human glioblastoma, we may expect further increasing interest in the research on its direct interactions with other ncRNAs regarding brain tumor pathology.

MiR-128-3P AS A POTENT MODULATOR OF THE IMMUNE MICROENVIRONMENT

In recent years, considerable efforts have been undertaken to determine and understand the role of microRNAs in regulating the immune response and microenvironment. Among other microRNAs, miR-128-3p was shown to be involved in immune and autoimmune processes pertaining to the brain.

Table 3. Summary of miR-128-3p deregulations and their effects on the modulation of the immune microenvironment in neurodegenerative diseases and malignancies of the human CNS

Disease		miR-128-3p deregulation	Function of miR-128
Neurodegenerative disorders	Alzheimer's disease	Up-regulated	Enhancement of amyloid beta degradation by monocytes
	Parkinson's disease	Up-regulated	Diminish in expression and secretion of IL-6 and IL-10, an increase of IL-12
	Multiple sclerosis	Up-regulated	Mediation of the Th2 into Th1 cytokine shift; inhibition of differentiation of Th2 and promotion of Th1 activity
Cancers	Melanoma	Up-regulated	Enhancement of DCs-mediated antitumor immunity
	Pancreatic adenocarcinoma	Up-regulated	Increase in the percentages of DCs, CD8+ T cells, and NKT
	Lymphoid malignancies	Up-regulated	A marker of lymphoid lineage differentiation; regulation of the survival and proliferation of thymocytes

Neurodegenerative diseases

A significantly increased level of miR-128-3p was found in naive $CD4^+$ T cells of patients with MS compared with healthy donors, demonstrating its previously unexplored roles in regulating the function of immune cells.[69,70] Overexpression of miR-128-3p in T cells reduced expression of IL-4, an effector cytokine of T helper cell type 2 (Th2). Consequently, the differentiation of Th2 cells was inhibited, while the pro-inflammatory activity of T helper type 1 (Th1) cells was promoted, resulting in an induced autoimmune response. Therefore, up-regulated miR-128-3p mediated the Th2 into Th1 cytokine shift by suppressing the development and activation of Th2 cells in patients with MS.[69,70] Overexpression of miR-128-3p diminished expression and secretion of IL-6 and IL-10 and increased IL-12, modifying the immune response in PD individuals.[62] Also, in AD patients, the expression of miR-128-3p was increased in lymphocytes and monocytes compared with healthy individuals. As a result, monocyte amyloid beta degradation was enhanced *in vitro*,[54] showing up-regulated miR-128-3p as a new potential target in AD.

Cancers

Several reports demonstrated the high level of miR-128-3p as a pro-inflammatory and anti-immunosuppressive factor in immune cells, potentially significantly enhancing antitumor immunity. MiR-128-3p was involved in the antitumor activity of dendritic cells (DCs) through inhibition of p38 expression and decrease in downstream levels of cytokines secreted by DCs in a melanoma mouse model.[176] Overexpression miR-128-3p in pancreatic adenocarcinoma was inversely correlated with CD47 (cluster of differentiation 47),[177] a cancer-associated antigen involved in the immune evasion of tumors.[178] Overexpression of miR-128-3p considerably increased the percentages of DCs, $CD8^+$ T cells, and natural killer T cells (NKT) within the tumor and spleen. Therefore, a significantly enhanced antitumor response was observed.[177] The high levels of miR-128-3p were also observed in thymocytes and distinguished the cells of lymphoid lineages from the myeloid lineages precursor cells in a mouse model of normal hematopoiesis and human lymphoid malignancies.[179]

Particular attention should also be paid to the potential role of miR-128-3p in regulating the immune microenvironment of brain tumors, especially glioblastoma. Several novel therapeutic strategies that aim to engage and activate patients' immune system against brain tumor cells were anticipated as promising, potentially game-changing strategies in the field of neuro-oncology. However, the immunosuppressive character of glioblastoma manifested by a "cold" microenvironment that results in poor immune cell infiltration,[180,181] concurrent with the fast progression of the disease, contributes to a short window for treatment opportunities. Therefore, the efficacy of immunotherapeutic approaches, including immune checkpoint blockade, effective in other solid tumors, such as lung cancer,[182,183] is considerably limited.

Summary of miR-128-3p as a potent modulator of the immune microenvironment

The studies on the role of miR-128-3p in neuropathologies clearly evidenced its potential utility in developing and enhancing immune-related therapies. High levels of miR-28 in some neurodegenerative disorders open avenues for genetic or pharmacologic targeting to induce immunosuppression. Conversely, replacing miR-128-3p would be effective for immune-boosting strategies in glioblastoma and, therefore, beneficial for patients. We summarized the role of miR-128-3p deregulations in modulating immune response and immune microenvironment in Table 3.

MiR-128-3P IN THE TREATMENT OF CNS PATHOLOGIES

MiR-128-3p as a therapeutic agent

The therapeutic use of oligonucleotides (microRNA inhibitors, mimics, siRNAs, and antisense oligonucleotides [ASOs]) is becoming increasingly widespread in treating CNS diseases. MicroRNA mimics refer to artificially created short double-stranded oligonucleotides that replicate microRNA precursors, which, upon their introduction into cells, are identified by the microRNA biogenesis machinery and undergo subsequent processing. MicroRNA inhibitors prevent the interaction between microRNA and the microRNA-induced silencing complex (miRISC) proteins and between the miRISC and its target mRNAs, thus preventing microRNA-mediated silencing of mRNA. Single-stranded ASOs target a specific RNA to block its translation into protein and/or trigger its degradation. Some ASOs were clinically approved for certain neuromuscular diseases and are currently in trials for other conditions.[184,185] The U.S. Food and Drug

Administration approved the first CNS-related oligonucleotide-based drug, Nusinersen, for treating spinal muscular atrophy caused by a survival motor neuron (SMN) protein deficiency. Nusinersen is designed to modify the splicing of the *SMN2* (Survival of motor neuron 2, centromeric) gene, increasing the production of functional SMN protein.[184,186,187] Diverse strategies in other CNS disease therapies utilize microRNA inhibitors,[188] mimics,[189] siRNAs,[190] and ASOs[191] to modulate treatment efficacy. For therapy to be effective, modifications of these molecules are often necessary to tailor them to the specific microenvironment in which they are intended to act. These novel nucleic acid modifications play a crucial role in enhancing the efficacy of microRNAs in molecular therapy strategies for CNS diseases.

In the case of miR-128-3p, given its involvement in neurodevelopment and gene expression regulation, these molecules hold promise for therapeutic strategies for CNS diseases. Dysregulated miR-128-3p in gliomas has led to investigations for potential therapeutic strategies. Preclinical studies using miR-128-3p mimics show promise in inhibiting GSC growth.[79] In CNS disorders like AD, miR-128-3p inhibitors may be versatile, targeting PPARG and potentially reducing amyloid beta levels.[53] Additionally, siRNA against miR-128-3p could be applied in neurodegenerative disorders like PD, offering a targeted approach to modulate disease-related pathways.[192,193]

Many modifications can be applied to microRNA inhibitors and mimics, improving their stability, permeability, and specificity. These include phosphorothioate,[194] 2′-O-methoxyethyl group,[195] locked nucleic acid,[17] fluorine derivatives, peptide nucleic acids,[196] and mixed modifications oligonucleotides. In the CNS, where enzymatic degradation is a concern, stabilizing modifications help prolong the half-life of therapeutic microRNAs.[197–199] Chemical modifications, such as 2′-sugar modifications like 2′-O-methyl and 2′-fluoro substitutions, enhance the stability[200,201] of microRNAs, making it more resistant to degradation by nucleases and increasing stability without impeding RISC recognition. Overall, these modifications contribute to prolonged circulation in the bloodstream and improved delivery to the CNS.[190]

Moreover, site-specific modifications within the microRNA sequence can enhance its binding affinity and specificity to target mRNAs implicated in CNS diseases, thus enforcing more precise and effective modulation of gene expression and reducing off-target effects.[202] Importantly, chemical modifications (e.g., 2′-O-methyl) can help mitigate the immunogenic response to synthetic microRNAs, ensuring that therapeutic molecules are well-tolerated and do not elicit adverse immune reactions in the CNS.[190] Finally, using conjugates, such as lipid nanoparticles or other delivery vehicles, facilitates the transport of modified microRNAs across the blood-brain barrier, a critical step in reaching the target cells within the CNS, thus promoting the efficient uptake and sustained therapeutic effects.[203,204]

Thus, through the deliberate use of chemical modifications, researchers aim to overcome challenges related to the delivery and functionality of microRNA therapeutics in the complex CNS environment. These modifications contribute to developing safer, more stable, and precise microRNA-based therapies for various CNS disorders.

While research showed that microRNA replacement or inhibition is, in fact, a promising therapeutic option, the current methods for microRNA modulation using oligonucleotides and gene therapies are challenging, particularly for neurological conditions, and none have yet been approved for clinical use. Recently, a new approach has been developed, which involves screening a biodiverse library of small molecule compounds to determine their ability to modulate hundreds of microRNAs. This method has shown promising results, with a dataset of 1,370 effective drug-like compounds providing a valuable resource for further microRNA-based drug discovery.[205]

Drug resistance is one of the major causes of failures of currently available cancer treatment strategies. MicroRNA-based strategies have gained attention as effective methods for sensitizing cancer cells because many microRNAs contribute to the increased resistance of cancer cells to standard treatments.[206,207] MicroRNAs possess several unique characteristics that make them desirable candidates for the potential development of pro-sensitization approaches and therapeutic agents. They are small molecules frequently conserved among species[208] that are remarkably stable in plasma and serum due to high resistance to RNase activity and degradation.[209] Moreover, microRNAs can re-arrange a genetic and molecular landscape in a cell-type-specific manner[130] due to the strict tissue and cell-type-specific distribution and functions of microRNAs,[210] meaning their targets in cancer and normal cells are different. We hypothesize that microRNAs that exert harmful effects on cancer cells may have, in fact, beneficial roles in non-malignant cells, including immune cells. The hypothesis is based on the observation that microRNAs often exhibit cell-type-specific functions and thus can regulate diverse biological processes in healthy and pathological tissue. Further research is thus warranted to prove this hypothesis and elucidate the mechanisms by which microRNAs differentially impact cancer and non-malignant cells.

MicroRNAs particularly enriched in the normal brain, such as miR-128-3p, are almost universally suppressed in glioblastoma cells, regardless of their cellular subtype.[45,126,130,211] This observation led to the development of the following strategy: microRNAs lost in tumor cells, once reintroduced, are detrimental to them while not harming normal, healthy cells. Crucially, many microRNAs coordinately target numerous factors from the same pathway, thus circumventing their physiological redundancy, e.g., miR-128-3p targeting several vital players of the PRC pathway.[77,130] Some functional studies provided several targets of miR-128-3p in cancer and immune cells,[126,130] thus supporting the rationale for its application to enhance antitumor and pro-immune activity.

The varying expression patterns of miR-128-3p in different neurological disorders suggest that its function is context-dependent and influenced by various cellular and environmental factors. Understanding

these context-specific roles is crucial for developing targeted therapies. Thus, reversing altered miR-128-3p expression, either up- or down-regulating it, can lead to unintended off-target effects due to its involvement in multiple cellular processes and interactions with various genes. Cell-type-specific delivery systems, such as nanoparticle encapsulation of microRNA mimics or inhibitors, would minimize off-target effects and improve therapeutic efficacy. Enforcing such cell-type selectivity would be critical when altering miR-128-3p expression. For instance, targeting dopamine neurons in PD disease without affecting other neuronal populations or immune cells in MS patients requires precise targeting strategies. Moreover, determining the optimal levels of miR-128-3p for therapeutic benefit is challenging due to the complex interplay with other microRNAs and the context-dependent nature of its function. Titration and dose optimization studies would be essential to identify the range required for therapeutic efficacy in different neurological disorders.

In conclusion, developing miR-128-based therapies requires a comprehensive understanding of its molecular mechanisms, regulatory networks, and context-specific functions in various neurological pathologies. While miR-128-3p holds promise as a therapeutic target, the complexity of its role in neurological disorders and the potential for off-target effects necessitate careful consideration, and further research is needed to elucidate these intricate relationships and guide the development of safe and effective therapies.

EV-mediated delivery of miR-128-3p

One of the most critical challenges for cancer molecular targeted therapy is the specific delivery of the therapeutic agent while avoiding its untimely degradation in the bloodstream or excretion. EVs, as the naturally secreted carriers of functional biomolecules (nucleic acids: genomic DNA, cDNA; RNAs: mRNA, microRNA, lncRNA; proteins and lipids)[212] have recently attracted much attention as potentially useful nano-tools for transferring therapeutic agents, including microRNAs. This is due to their advantageous features, such as small size, the ability to enclose numerous molecules and particles, and the scaled-up production of specific engineered RNA-based agents. Over recent decades, EVs have been identified as vehicles of therapeutic agents with clinical relevance owing to their ability to transport numerous biomolecules between cells and tissues. While EVs exhibit some tissue or cell tropism,[213,214] their targeting specificity can be less precise than synthetic delivery vehicles. Moreover, the specific targeting properties of EVs are influenced by various factors, including the cell of origin, the presence of surface molecules, and the cargo they carry.

Incorporating targeting ligands, peptides, or aptamers is a promising strategy to improve EVs' targeting efficacy for therapeutic applications. These modifications can enhance the ability of EVs to selectively bind to specific cell types or tissues, thereby reducing off-target delivery and increasing the therapeutic potential of the encapsulated cargo,[215,216] including in cancer[217,218] and CNS pathologies.[219,220] More importantly, EVs originating from non-malignant cells do not propagate pro-tumorigenic signals to cancer cells and are not toxic to normal cells and tissues.[69,77,126,130,221] The ability to prepare a large number of EVs carrying therapeutic microRNAs for cancer patients within a short time after surgery is also crucial, as the fast progression of the disease contributes to very limited opportunities for effective therapy. Furthermore, molecules encapsulated within blood-circulating EVs may also serve as diagnostic, prognostic, and predictive biomarkers.[222,223] Several studies demonstrated the feasibility of EV-mediated transfer of specific microRNAs in the modulation of the tumor microenvironment and, consequently, its effect on the tumor growth, progression, and response to standard treatment modalities in breast cancer,[224,225] hepatocellular carcinomas,[226,227] and lung cancer cells *in vitro*.[228–230]

The delivery of therapeutic RNA molecules (siRNA or microRNA) encapsulated within the lipid or lipid-like carrier[231,232] into the cells or tissues was proven to be effective in infectious diseases and tumors, as shown in many preclinical studies[233–235] and clinical trials (reviewed by Kaczmarek et al.[236]). We have recently witnessed the development and successful implementation of anti-COVID-19 vaccines based on mRNA into the global pharmaceutical market. These vaccines are based on synthetic lipid nanoparticles designed to encapsulate and protect mRNA molecules and facilitate their cell entry, and their approval for clinical usage has been instrumental in developing successful COVID-19 vaccines effectively. Although not based on EVs, they nevertheless highlight the ability of lipid vesicles to maintain the functionality of enclosed RNAs and effectively boost immune response *in vivo*.

Over recent decades, EVs have been identified as vehicles of therapeutic agents with clinical relevance owing to their ability to transport numerous biomolecules between cells and tissues and, therefore, as nano-carriers for targeted delivery. Moreover, EV-based delivery of microRNAs with proven anti-oncogenic and immune-enhancing properties, such as miR-128-3p, represents a potential experimental strategy for developing the therapy against glioblastoma and human cancer in general. However, significant efforts are still needed to broaden our knowledge on the heterogeneity of EVs and EV-enclosed microRNAs as therapeutic agents to translate this approach into clinical practice. Optimization and standardization of the protocols for the isolation and characterization of EVs, including the acquirement of large-scale yield and high purity of EVs, are necessary to guide future research and establish the standards for medical applications. Also, several other aspects should be prioritized, such as biodistribution, pharmacodynamics, and pharmacokinetics of the administered EV-microRNA complexes *in vivo*.

SUMMARY

This review discusses the importance of miR-128-3p in healthy human CNS and its pathologies (Figure 5). Up-regulated expression of miR-128-3p within the CNS implicates its critical role in developing and maintaining the brain's biological and physiological functions. Consequently, the deregulations of miR-128-3p are strongly

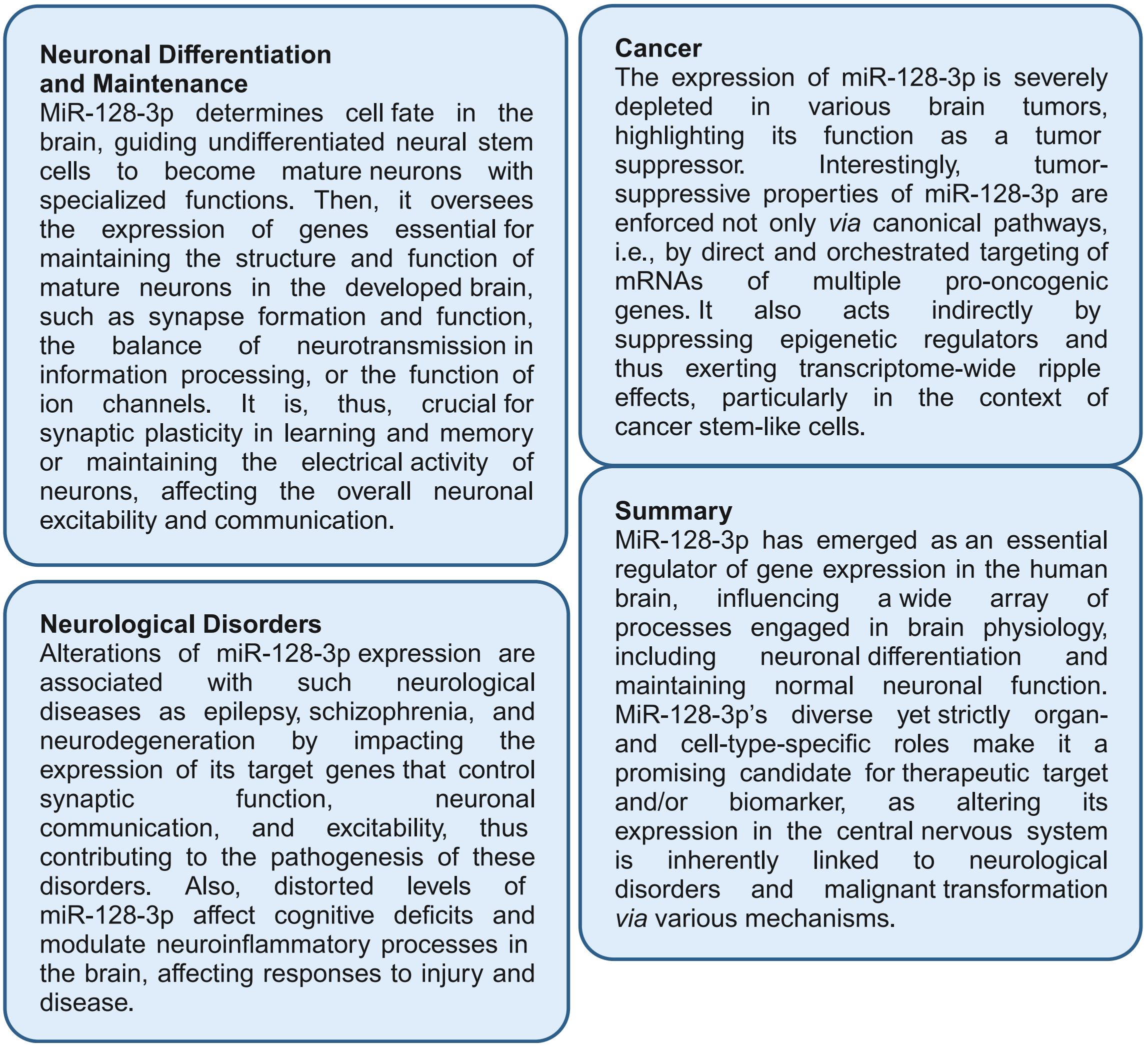

Figure 5. The implications of miR-128-3p for the brain function

The boxes summarize the crucial roles of miR-128-3p in several aspects of CNS function, such as differentiation and maintenance of neurons, pathogenesis of brain tumors, and neurological diseases.

related to numerous brain and spinal cord pathologies, including neurodegenerative disorders, neurological damage, injuries, and malignant tumors. The increasing interest in miR-128-3p and its interference with other ncRNAs and several signaling pathways suggests the high clinical relevance of miR-128-3p and makes it a good candidate for therapeutic intervention. Due to its high expression in neurodegenerative scenarios, miR-128-3p may be genetically or pharmacologically targeted to induce immunosuppression, as opposed to immune-boosting strategies, due to its dramatically reduced level in malignant brain tumors compared with normal brain tissue. The opposite correlation of miR-128-3p expression—loss in malignant brain tumors and prominent up-regulation in neurodegeneration—clearly emphasizes the importance of its balanced expression, suggesting potential reverse therapeutic opportunities in both pathological states. The accumulating evidence from preclinical and clinical studies also suggests that delivering microRNAs via EVs is a feasible and promising novel approach for increasing the sensitivity of cancer cells to cytotoxic agents while enhancing their immunogenicity. Therefore, we expect further growing interest in the potential application of EV-based transfer of specific therapeutic RNAs, e.g., miR-128-3p, in treating human cancers, including brain tumors.

SUPPLEMENTAL INFORMATION

Supplemental information can be found online at https://doi.org/10.1016/j.omtn.2024.102141.

ACKNOWLEDGMENTS

Supported by National Science Centre Poland grants: 2021/41/N/NZ6/02682 (to K.K.), 2018/29/B/NZ1/01016 and 2022/45/B/NZ5/04226 (to A.B.), 2020/39/B/NZ5/02893 and 2021/41/B/NZ6/02436

(to J.G.); and by Polish National Agency for Academic Exchange grant: PPN/PPO/2019/1/00001 (to J.G.).

AUTHOR CONTRIBUTIONS

Conceptualization, A.B. and J.G.; Data curation, K.K. and S.K.K.; Formal Analysis, K.K. and S.K.K.; Funding acquisition, K.K., A.B., and J.G.; Project administration, K.K., A.B., and J.G.; Resources, K.K. and S.K.K.; Software, K.K. and S.K.K.; Supervision, A.B. and J.G.; Visualization, K.K. and S.K.K.; Writing–original draft and figures preparation, K.K. and S.K.K.; Writing–review and editing, K.K., S.K.K, A.B., and J.G. All authors have read and agreed to the published version of the manuscript.

DECLARATION OF INTERESTS

The authors declare no competing interests.

REFERENCES

1. MacFarlane, L.-A., and Murphy, P.R. (2010). MicroRNA: Biogenesis, Function and Role in Cancer. Curr. Genomics *11*, 537–561. https://doi.org/10.2174/138920210793175895.
2. Oliveto, S., Mancino, M., Manfrini, N., and Biffo, S. (2017). Role of microRNAs in translation regulation and cancer. World J. Biol. Chem. *8*, 45–56. https://doi.org/10.4331/wjbc.v8.i1.45.
3. O'Brien, J., Hayder, H., Zayed, Y., and Peng, C. (2018). Overview of MicroRNA Biogenesis, Mechanisms of Actions, and Circulation. Front. Endocrinol. *9*, 402. https://doi.org/10.3389/fendo.2018.00402.
4. Godlewski, J., Krichevsky, A.M., Johnson, M.D., Chiocca, E.A., and Bronisz, A. (2015). Belonging to a network–microRNAs, extracellular vesicles, and the glioblastoma microenvironment. Neuro Oncol. *17*, 652–662. https://doi.org/10.1093/neuonc/nou292.
5. Kinoshita, T., Yip, K.W., Spence, T., and Liu, F.-F. (2017). MicroRNAs in extracellular vesicles: potential cancer biomarkers. J. Hum. Genet. *62*, 67–74. https://doi.org/10.1038/jhg.2016.87.
6. Godlewski, J., Lenart, J., and Salinska, E. (2019). MicroRNA in Brain pathology: Neurodegeneration the Other Side of the Brain Cancer. Noncoding. RNA *5*, 20. https://doi.org/10.3390/ncrna5010020.
7. Condrat, C.E., Thompson, D.C., Barbu, M.G., Bugnar, O.L., Boboc, A., Cretoiu, D., Suciu, N., Cretoiu, S.M., and Voinea, S.C. (2020). miRNAs as Biomarkers in Disease: Latest Findings Regarding Their Role in Diagnosis and Prognosis. Cells *9*, 276. https://doi.org/10.3390/cells9020276.
8. Lee, R.C., Feinbaum, R.L., and Ambros, V. (1993). The C. elegans heterochronic gene lin-4 encodes small RNAs with antisense complementarity to lin-14. Cell *75*, 843–854. https://doi.org/10.1016/0092-8674(93)90529-y.
9. Pasquinelli, A.E., Reinhart, B.J., Slack, F., Martindale, M.Q., Kuroda, M.I., Maller, B., Hayward, D.C., Ball, E.E., Degnan, B., Müller, P., et al. (2000). Conservation of the sequence and temporal expression of let-7 heterochronic regulatory RNA. Nature *408*, 86–89. https://doi.org/10.1038/35040556.
10. Calin, G.A., Dumitru, C.D., Shimizu, M., Bichi, R., Zupo, S., Noch, E., Aldler, H., Rattan, S., Keating, M., Rai, K., et al. (2002). Frequent deletions and down-regulation of micro- RNA genes miR15 and miR16 at 13q14 in chronic lymphocytic leukemia. Proc. Natl. Acad. Sci. USA *99*, 15524–15529. https://doi.org/10.1073/pnas.242606799.
11. Volinia, S., Calin, G.A., Liu, C.-G., Ambs, S., Cimmino, A., Petrocca, F., Visone, R., Iorio, M., Roldo, C., Ferracin, M., et al. (2006). A microRNA expression signature of human solid tumors defines cancer gene targets. Proc. Natl. Acad. Sci. USA *103*, 2257–2261. https://doi.org/10.1073/pnas.0510565103.
12. Lukiw, W.J. (2007). Micro-RNA speciation in fetal, adult and Alzheimer's disease hippocampus. Neuroreport *18*, 297–300. https://doi.org/10.1097/WNR.0b013e3280148e8b.
13. Lawrie, C.H., Gal, S., Dunlop, H.M., Pushkaran, B., Liggins, A.P., Pulford, K., Banham, A.H., Pezzella, F., Boultwood, J., Wainscoat, J.S., et al. (2008). Detection of elevated levels of tumour-associated microRNAs in serum of patients with diffuse large B-cell lymphoma. Br. J. Haematol. *141*, 672–675. https://doi.org/10.1111/j.1365-2141.2008.07077.x.
14. Lindow, M., and Kauppinen, S. (2012). Discovering the first microRNA-targeted drug. J. Cell Biol. *199*, 407–412. https://doi.org/10.1083/jcb.201208082.
15. ENCODE Project Consortium (2012). An integrated encyclopedia of DNA elements in the human genome. Nature *489*, 57–74. https://doi.org/10.1038/nature11247.
16. Melo, S.A., Sugimoto, H., O'Connell, J.T., Kato, N., Villanueva, A., Vidal, A., Qiu, L., Vitkin, E., Perelman, L.T., Melo, C.A., et al. (2014). Cancer exosomes perform cell-independent microRNA biogenesis and promote tumorigenesis. Cancer Cell *26*, 707–721. https://doi.org/10.1016/j.ccell.2014.09.005.
17. Cheng, C.J., Bahal, R., Babar, I.A., Pincus, Z., Barrera, F., Liu, C., Svoronos, A., Braddock, D.T., Glazer, P.M., Engelman, D.M., et al. (2015). MicroRNA silencing for cancer therapy targeted to the tumour microenvironment. Nature *518*, 107–110. https://doi.org/10.1038/nature13905.
18. Lorenzi, L., Chiu, H.-S., Avila Cobos, F., Gross, S., Volders, P.-J., Cannoodt, R., Nuytens, J., Vanderheyden, K., Anckaert, J., Lefever, S., et al. (2021). The RNA Atlas expands the catalog of human non-coding RNAs. Nat. Biotechnol. *39*, 1453–1465. https://doi.org/10.1038/s41587-021-00936-1.
19. Yamaguchi, S., Naganuma, M., Nishizawa, T., Kusakizako, T., Tomari, Y., Nishimasu, H., and Nureki, O. (2022). Structure of the Dicer-2-R2D2 heterodimer bound to a small RNA duplex. Nature *607*, 393–398. https://doi.org/10.1038/s41586-022-04790-2.
20. Meister, G. (2023). MicroRNA uses a gym to get fit for cuts by Dicer enzyme. Nature *615*, 218–219. https://doi.org/10.1038/d41586-023-00478-3.
21. Saliminejad, K., Khorram Khorshid, H.R., Soleymani Fard, S., and Ghaffari, S.H. (2019). An overview of microRNAs: Biology, functions, therapeutics, and analysis methods. J. Cell. Physiol. *234*, 5451–5465. https://doi.org/10.1002/jcp.27486.
22. Shao, N.-Y., Hu, H.Y., Yan, Z., Xu, Y., Hu, H., Menzel, C., Li, N., Chen, W., and Khaitovich, P. (2010). Comprehensive survey of human brain microRNA by deep sequencing. BMC Genom. *11*, 409. https://doi.org/10.1186/1471-2164-11-409.
23. Kim, V.N., Han, J., and Siomi, M.C. (2009). Biogenesis of small RNAs in animals. Nat. Rev. Mol. Cell Biol. *10*, 126–139. https://doi.org/10.1038/nrm2632.
24. Zeng, Y. (2006). Principles of micro-RNA production and maturation. Oncogene *25*, 6156–6162. https://doi.org/10.1038/sj.onc.1209908.
25. Monteys, A.M., Spengler, R.M., Wan, J., Tecedor, L., Lennox, K.A., Xing, Y., and Davidson, B.L. (2010). Structure and activity of putative intronic miRNA promoters. RNA N. Y. N *16*, 495–505. https://doi.org/10.1261/rna.1731910.
26. Kim, V.N. (2005). MicroRNA biogenesis: coordinated cropping and dicing. Nat. Rev. Mol. Cell Biol. *6*, 376–385. https://doi.org/10.1038/nrm1644.
27. Sjöstedt, E., Zhong, W., Fagerberg, L., Karlsson, M., Mitsios, N., Adori, C., Oksvold, P., Edfors, F., Limiszewska, A., Hikmet, F., et al. (2020). An atlas of the protein-coding genes in the human, pig, and mouse brain. Science *367*, eaay5947. https://doi.org/10.1126/science.aay5947.
28. Liu, B., Shyr, Y., Cai, J., and Liu, Q. (2018). Interplay between miRNAs and host genes and their role in cancer. Brief. Funct. Genomics *18*, 255–266. https://doi.org/10.1093/bfgp/elz002.
29. Tan, C.L., Plotkin, J.L., Venø, M.T., von Schimmelmann, M., Feinberg, P., Mann, S., Handler, A., Kjems, J., Surmeier, D.J., O'Carroll, D., et al. (2013). MicroRNA-128 Governs Neuronal Excitability and Motor Behavior in Mice. Science *342*, 1254–1258. https://doi.org/10.1126/science.1244193.
30. Keller, A., Gröger, L., Tschernig, T., Solomon, J., Laham, O., Schaum, N., Wagner, V., Kern, F., Schmartz, G.P., Li, Y., et al. (2022). miRNATissueAtlas2: an update to the human miRNA tissue atlas. Nucleic Acids Res. *50*, D211–D221. https://doi.org/10.1093/nar/gkab808.
31. Smirnova, L., Gräfe, A., Seiler, A., Schumacher, S., Nitsch, R., and Wulczyn, F.G. (2005). Regulation of miRNA expression during neural cell specification. Eur. J. Neurosci. *21*, 1469–1477. https://doi.org/10.1111/j.1460-9568.2005.03978.x.

32. He, M., Liu, Y., Wang, X., Zhang, M.Q., Hannon, G.J., and Huang, Z.J. (2012). Cell-Type-Based Analysis of MicroRNA Profiles in the Mouse Brain. Neuron *73*, 35–48. https://doi.org/10.1016/j.neuron.2011.11.010.

33. Zhang, W., Kim, P.J., Chen, Z., Lokman, H., Qiu, L., Zhang, K., Rozen, S.G., Tan, E.K., Je, H.S., and Zeng, L. (2016). MiRNA-128 regulates the proliferation and neurogenesis of neural precursors by targeting PCM1 in the developing cortex. Elife *5*, e11324. https://doi.org/10.7554/eLife.11324.

34. Shu, P., Wu, C., Ruan, X., Liu, W., Hou, L., Fu, H., Wang, M., Liu, C., Zeng, Y., Chen, P., et al. (2019). Opposing Gradients of MicroRNA Expression Temporally Pattern Layer Formation in the Developing Neocortex. Dev. Cell *49*, 764–785.e4. https://doi.org/10.1016/j.devcel.2019.04.017.

35. Mellios, N., and Sur, M. (2012). The Emerging Role of microRNAs in Schizophrenia and Autism Spectrum Disorders. Front. Psychiatry 3, 39.

36. Ching, A.-S., and Ahmad-Annuar, A. (2015). A Perspective on the Role of microRNA-128 Regulation in Mental and Behavioral Disorders. Front. Cell. Neurosci. 9, 465.

37. Franzoni, E., Booker, S.A., Parthasarathy, S., Rehfeld, F., Grosser, S., Srivatsa, S., Fuchs, H.R., Tarabykin, V., Vida, I., and Wulczyn, F.G. (2015). miR-128 regulates neuronal migration, outgrowth and intrinsic excitability via the intellectual disability gene Phf6. Elife *4*, e04263. https://doi.org/10.7554/eLife.04263.

38. Jahani-Asl, A., Cheng, C., Zhang, C., and Bonni, A. (2016). Pathogenesis of Börjeson-Forssman-Lehmann syndrome: Insights from PHF6 function. Neurobiol. Dis. *96*, 227–235. https://doi.org/10.1016/j.nbd.2016.09.011.

39. Feng, Y., Yang, H., Yue, Y., and Tian, F. (2020). MicroRNAs and target genes in epileptogenesis. Epilepsia *61*, 2086–2096. https://doi.org/10.1111/epi.16687.

40. Chen, P.-H., Cheng, C.-H., Shih, C.-M., Ho, K.-H., Lin, C.-W., Lee, C.-C., Liu, A.-J., Chang, C.-K., and Chen, K.-C. (2016). The Inhibition of microRNA-128 on IGF-1-Activating mTOR Signaling Involves in Temozolomide-Induced Glioma Cell Apoptotic Death. PLoS One *11*, e0167096. https://doi.org/10.1371/journal.pone.0167096.

41. Yao, M., Li, S., Wu, X., Diao, S., Zhang, G., He, H., Bian, L., and Lu, Y. (2018). Cellular origin of glioblastoma and its implication in precision therapy. Cell. Mol. Immunol. *15*, 737–739. https://doi.org/10.1038/cmi.2017.159.

42. Lee, J.H., Lee, J.E., Kahng, J.Y., Kim, S.H., Park, J.S., Yoon, S.J., Um, J.-Y., Kim, W.K., Lee, J.-K., Park, J., et al. (2018). Human glioblastoma arises from subventricular zone cells with low-level driver mutations. Nature *560*, 243–247. https://doi.org/10.1038/s41586-018-0389-3.

43. Kim, H.J., Park, J.W., and Lee, J.H. (2020). Genetic Architectures and Cell-of-Origin in Glioblastoma. Front. Oncol. 10, 615400.

44. Shafi, O., and Siddiqui, G. (2022). Tracing the origins of glioblastoma by investigating the role of gliogenic and related neurogenic genes/signaling pathways in GBM development: a systematic review. World J. Surg. Oncol. *20*, 146. https://doi.org/10.1186/s12957-022-02602-5.

45. Bronisz, A., Rooj, A.K., Krawczyński, K., Peruzzi, P., Salińska, E., Nakano, I., Purow, B., Chiocca, E.A., and Godlewski, J. (2020). The nuclear DICER-circular RNA complex drives the deregulation of the glioblastoma cell microRNAome. Sci. Adv. *6*, eabc0221. https://doi.org/10.1126/sciadv.abc0221.

46. Szczepaniak, A., Bronisz, A., and Godlewski, J. (2023). Circular RNAs—New Kids on the Block in Cancer Pathophysiology and Management. Cells *12*, 552. https://doi.org/10.3390/cells12040552.

47. Men, Y., Ye, L., Risgaard, R.D., Promes, V., Zhao, X., Paukert, M., and Yang, Y. (2020). Astroglial FMRP deficiency cell-autonomously up-regulates miR-128 and disrupts developmental astroglial mGluR5 signaling. Proc. Natl. Acad. Sci. *117*, 25092–25103. https://doi.org/10.1073/pnas.2014080117.

48. Rahmani, S., Kadkhoda, S., and Ghafouri-Fard, S. (2022). Synaptic plasticity and depression: the role of miRNAs dysregulation. Mol. Biol. Rep. *49*, 9759–9765. https://doi.org/10.1007/s11033-022-07461-7.

49. Petry, S., Keraudren, R., Nateghi, B., Loiselle, A., Pircs, K., Jakobsson, J., Sephton, C., Langlois, M., St-Amour, I., and Hébert, S.S. (2022). Widespread alterations in microRNA biogenesis in human Huntington's disease putamen. Acta Neuropathol. Commun. *10*, 106. https://doi.org/10.1186/s40478-022-01407-7.

50. Pang, Y., Luo, D., and Wang, S. (2022). miR-128-3p inhibits the inflammation by targeting MAPK6 in penicillin-induced astrocytes. Neuroreport *33*, 742–749. https://doi.org/10.1097/WNR.0000000000001840.

51. Blair, M.A., Abou-Khalil, B., Crunk, A., Haines, J.L., and Hedera, P. (2011). A new locus for autosomal dominant generalized epilepsy associated with mild mental retardation on chromosome 3p. Epilepsia *52*, 993–999. https://doi.org/10.1111/j.1528-1167.2011.03059.x.

52. Yuan, Y., Xiang, W., Yanhui, L., Ruofei, L., Yunhe, M., Jiewen, L., and Qing, M. (2016). Dysregulation of microRNA-128 expression in WHO grades 2 glioma is associated with glioma-associated epilepsy: Down-regulation of miR-128 induces glioma-associated seizure. Epilepsy Res. *127*, 6–11. https://doi.org/10.1016/j.eplepsyres.2016.08.005.

53. Liu, Y., Zhang, Y., Liu, P., Bai, H., Li, X., Xiao, J., Yuan, Q., Geng, S., Yin, H., Zhang, H., et al. (2019). MicroRNA-128 knockout inhibits the development of Alzheimer's disease by targeting PPARγ in mouse models. Eur. J. Pharmacol. *843*, 134–144. https://doi.org/10.1016/j.ejphar.2018.11.004.

54. Geng, L., Zhang, T., Liu, W., and Chen, Y. (2018). Inhibition of miR-128 Abates Aβ-Mediated Cytotoxicity by Targeting PPAR-γ via NF-κB Inactivation in Primary Mouse Cortical Neurons and Neuro2a Cells. Yonsei Med. J. *59*, 1096–1106. https://doi.org/10.3349/ymj.2018.59.9.1096.

55. Ahuja, C.S., Wilson, J.R., Nori, S., Kotter, M.R.N., Druschel, C., Curt, A., and Fehlings, M.G. (2017). Traumatic spinal cord injury. Nat. Rev. Dis. Primers *3*, 17018. https://doi.org/10.1038/nrdp.2017.18.

56. Anjum, A., Yazid, M.D., Fauzi Daud, M., Idris, J., Ng, A.M.H., Selvi Naicker, A., Ismail, O.H.R., Athi Kumar, R.K., and Lokanathan, Y. (2020). Spinal Cord Injury: Pathophysiology, Multimolecular Interactions, and Underlying Recovery Mechanisms. Int. J. Mol. Sci. *21*, 7533. https://doi.org/10.3390/ijms21207533.

57. Liu, R., Peng, Z., Zhang, Y., Li, R., and Wang, Y. (2021). Upregulation of miR-128 inhibits neuronal cell apoptosis following spinal cord injury via FasL downregulation by repressing ULK1. Mol. Med. Rep. *24*, 667. https://doi.org/10.3892/mmr.2021.12306.

58. Zhu, Y., Yu, F., Jiao, Y., Feng, J., Tang, W., Yao, H., Gong, C., Chen, J., Su, F., Zhang, Y., and Song, E. (2011). Reduced miR-128 in breast tumor-initiating cells induces chemotherapeutic resistance via Bmi-1 and ABCC5. Clin. Cancer Res. *17*, 7105–7115. https://doi.org/10.1158/1078-0432.CCR-11-0071.

59. Zhang, X., Zhang, Y., Cai, W., Liu, Y., Liu, H., Zhang, Z., and Su, Z. (2020). MicroRNA-128-3p Alleviates Neuropathic Pain Through Targeting ZEB1. Neurosci. Lett. *729*, 134946. https://doi.org/10.1016/j.neulet.2020.134946.

60. Kalia, L.V., and Lang, A.E. (2015). Parkinson's disease. Lancet Lond. Engl. *386*, 896–912. https://doi.org/10.1016/S0140-6736(14)61393-3.

61. Simon, D.K., Tanner, C.M., and Brundin, P. (2020). Parkinson Disease Epidemiology, Pathology, Genetics, and Pathophysiology. Clin. Geriatr. Med. *36*, 1–12. https://doi.org/10.1016/j.cger.2019.08.002.

62. Zhou, L., Yang, L., Li, Y.J., Mei, R., Yu, H.L., Gong, Y., Du, M.Y., and Wang, F. (2018). MicroRNA-128 Protects Dopamine Neurons from Apoptosis and Upregulates the Expression of Excitatory Amino Acid Transporter 4 in Parkinson's Disease by Binding to AXIN1. Cell. Physiol. Biochem. *51*, 2275–2289. https://doi.org/10.1159/000495872.

63. Zhang, G., Chen, L., Liu, J., Jin, Y., Lin, Z., Du, S., Fu, Z., Chen, T., Qin, Y., Sui, F., and Jiang, Y. (2020). HIF-1α/microRNA-128-3p axis protects hippocampal neurons from apoptosis via the *Axin1*-mediated Wnt/β-catenin signaling pathway in Parkinson's disease models. Aging *12*, 4067–4081. https://doi.org/10.18632/aging.102636.

64. Mendelson, S.J., and Prabhakaran, S. (2021). Diagnosis and Management of Transient Ischemic Attack and Acute Ischemic Stroke: A Review. JAMA *325*, 1088–1098. https://doi.org/10.1001/jama.2020.26867.

65. Feske, S.K. (2021). Ischemic Stroke. Am. J. Med. *134*, 1457–1464. https://doi.org/10.1016/j.amjmed.2021.07.027.

66. Liu, P., Han, Z., Ma, Q., Liu, T., Wang, R., Tao, Z., Li, G., Li, F., Zhang, S., Li, L., et al. (2019). Upregulation of MicroRNA-128 in the Peripheral Blood of Acute Ischemic Stroke Patients is Correlated with Stroke Severity Partially through Inhibition of Neuronal Cell Cycle Reentry. Cell Transplant. *28*, 839–850. https://doi.org/10.1177/0963689719846848.

67. McGinley, M.P., Goldschmidt, C.H., and Rae-Grant, A.D. (2021). Diagnosis and Treatment of Multiple Sclerosis: A Review. JAMA *325*, 765–779. https://doi.org/10.1001/jama.2020.26858.

68. Olek, M.J. (2021). Multiple Sclerosis. Ann. Intern. Med. *174*, ITC81–ITC96. https://doi.org/10.7326/AITC202106150.

69. Guerau-de-Arellano, M., Smith, K.M., Godlewski, J., Liu, Y., Winger, R., Lawler, S.E., Whitacre, C.C., Racke, M.K., and Lovett-Racke, A.E. (2011). Micro-RNA dysregulation in multiple sclerosis favours pro-inflammatory T-cell-mediated autoimmunity. Brain *134*, 3578–3589. https://doi.org/10.1093/brain/awr262.

70. Severin, M.E., Lee, P.W., Liu, Y., Selhorst, A.J., Gormley, M.G., Pei, W., Yang, Y., Guerau-de-Arellano, M., Racke, M.K., and Lovett-Racke, A.E. (2016). MicroRNAs targeting TGFβ signalling underlie the regulatory T cell defect in multiple sclerosis. Brain *139*, 1747–1761. https://doi.org/10.1093/brain/aww084.

71. Wang, Q., Li, P., Li, A., Jiang, W., Wang, H., Wang, J., and Xie, K. (2012). Plasma specific miRNAs as predictive biomarkers for diagnosis and prognosis of glioma. J. Exp. Clin. Cancer Res. *31*, 97. https://doi.org/10.1186/1756-9966-31-97.

72. Sun, J., Liao, K., Wu, X., Huang, J., Zhang, S., and Lu, X. (2015). Serum microRNA-128 as a biomarker for diagnosis of glioma. Int. J. Clin. Exp. Med. 8, 456–463.

73. Lages, E., Guttin, A., El Atifi, M., Ramus, C., Ipas, H., Dupré, I., Rolland, D., Salon, C., Godfraind, C., deFraipont, F., et al. (2011). MicroRNA and Target Protein Patterns Reveal Physiopathological Features of Glioma Subtypes. PLoS One *6*, e20600. https://doi.org/10.1371/journal.pone.0020600.

74. Palumbo, T., Faucz, F.R., Azevedo, M., Xekouki, P., Iliopoulos, D., and Stratakis, C.A. (2013). Functional screen analysis reveals miR-26b and miR-128 as central regulators of pituitary somatomammotrophic tumor growth through activation of the PTEN–AKT pathway. Oncogene *32*, 1651–1659. https://doi.org/10.1038/onc.2012.190.

75. Eguía-Aguilar, P., Pérezpeña-Díazconti, M., Benadón-Darszon, E., Chico-Ponce de León, F., Gordillo-Domínguez, L., Torres-García, S., Sadowinski-Pine, S., and Arenas-Huertero, F. (2014). Reductions in the expression of miR-124-3p, miR-128-1, and miR-221-3p in pediatric astrocytomas are related to high-grade supratentorial, and recurrent tumors in Mexican children. Childs Nerv. Syst. *30*, 1173–1181. https://doi.org/10.1007/s00381-014-2416-5.

76. Xu, J., Liu, Y., Guo, S., Ma, S., Xiao, L., Wei, N., and Xue, R. (2016). Expression Profile of MiR-128 in the Astrocytoma Patients and Cell Lines. Mol. Neurobiol. *53*, 4631–4637. https://doi.org/10.1007/s12035-015-9401-1.

77. Peruzzi, P., Bronisz, A., Nowicki, M.O., Wang, Y., Ogawa, D., Price, R., Nakano, I., Kwon, C.-H., Hayes, J., Lawler, S.E., et al. (2013). MicroRNA-128 coordinately targets Polycomb Repressor Complexes in glioma stem cells. Neuro Oncol. *15*, 1212–1224. https://doi.org/10.1093/neuonc/not055.

78. Zhou, Q., Liu, J., Quan, J., Liu, W., Tan, H., and Li, W. (2018). MicroRNAs as potential biomarkers for the diagnosis of glioma: A systematic review and meta-analysis. Cancer Sci. *109*, 2651–2659. https://doi.org/10.1111/cas.13714.

79. Zhao, C., Guo, R., Guan, F., Ma, S., Li, M., Wu, J., Liu, X., Li, H., and Yang, B. (2020). MicroRNA-128-3p Enhances the Chemosensitivity of Temozolomide in Glioblastoma by Targeting c-Met and EMT. Sci. Rep. *10*, 9471. https://doi.org/10.1038/s41598-020-65331-3.

80. Louis, D.N., Perry, A., Wesseling, P., Brat, D.J., Cree, I.A., Figarella-Branger, D., Hawkins, C., Ng, H.K., Pfister, S.M., Reifenberger, G., et al. (2021). The 2021 WHO Classification of Tumors of the Central Nervous System: a summary. Neuro Oncol. *23*, 1231–1251. https://doi.org/10.1093/neuonc/noab106.

81. Ostrom, Q.T., Gittleman, H., Fulop, J., Liu, M., Blanda, R., Kromer, C., Wolinsky, Y., Kruchko, C., and Barnholtz-Sloan, J.S. (2015). CBTRUS statistical report: primary brain and central nervous system tumors diagnosed in the united states in 2008-2012. Neuro Oncol. *17*, iv1–iv62. https://doi.org/10.1093/neuonc/nov189.

82. Venkataraman, S., Alimova, I., Fan, R., Harris, P., Foreman, N., and Vibhakar, R. (2010). MicroRNA 128a Increases Intracellular ROS Level by Targeting Bmi-1 and Inhibits Medulloblastoma Cancer Cell Growth by Promoting Senescence. PLoS One *5*, e10748. https://doi.org/10.1371/journal.pone.0010748.

83. Molitch, M.E. (2017). Diagnosis and Treatment of Pituitary Adenomas: A Review. JAMA *317*, 516–524. https://doi.org/10.1001/jama.2016.19699.

84. Rusetska, N., Kober, P., Król, S.K., Boresowicz, J., Maksymowicz, M., Kunicki, J., Bonicki, W., and Bujko, M. (2021). Invasive and Noninvasive Nonfunctioning Gonadotroph Pituitary Tumors Differ in DNA Methylation Level of LINE-1 Repetitive Elements. J. Clin. Med. *10*, 560. https://doi.org/10.3390/jcm10040560.

85. Tritos, N.A., and Miller, K.K. (2023). Diagnosis and Management of Pituitary Adenomas: A Review. JAMA *329*, 1386–1398. https://doi.org/10.1001/jama.2023.5444.

86. Patel, A.P., Tirosh, I., Trombetta, J.J., Shalek, A.K., Gillespie, S.M., Wakimoto, H., Cahill, D.P., Nahed, B.V., Curry, W.T., Martuza, R.L., et al. (2014). Single-cell RNA-seq highlights intratumoral heterogeneity in primary glioblastoma. Science *344*, 1396–1401. https://doi.org/10.1126/science.1254257.

87. Soeda, A., Hara, A., Kunisada, T., Yoshimura, S.i., Iwama, T., and Park, D.M. (2015). The Evidence of Glioblastoma Heterogeneity. Sci. Rep. *5*, 7979. https://doi.org/10.1038/srep07979.

88. Bianco, J., Bastiancich, C., Jankovski, A., des Rieux, A., Préat, V., and Danhier, F. (2017). On glioblastoma and the search for a cure: where do we stand? Cell. Mol. Life Sci. *74*, 2451–2466. https://doi.org/10.1007/s00018-017-2483-3.

89. Roura, A.-J., Gielniewski, B., Pilanc, P., Szadkowska, P., Maleszewska, M., Krol, S.K., Czepko, R., Kaspera, W., Wojtas, B., and Kaminska, B. (2021). Identification of the immune gene expression signature associated with recurrence of high-grade gliomas. J. Mol. Med. *99*, 241–255. https://doi.org/10.1007/s00109-020-02005-7.

90. Singh, S.K., Clarke, I.D., Terasaki, M., Bonn, V.E., Hawkins, C., Squire, J., and Dirks, P.B. (2003). Identification of a Cancer Stem Cell in Human Brain Tumors. Cancer Res. *63*, 5821–5828.

91. Lathia, J.D., Mack, S.C., Mulkearns-Hubert, E.E., Valentim, C.L.L., and Rich, J.N. (2015). Cancer stem cells in glioblastoma. Genes Dev. *29*, 1203–1217. https://doi.org/10.1101/gad.261982.115.

92. Yi, Y., Hsieh, I.-Y., Huang, X., Li, J., and Zhao, W. (2016). Glioblastoma Stem-Like Cells: Characteristics, Microenvironment, and Therapy. Front. Pharmacol. *7*, 477.

93. Król, S.K., Kaczmarczyk, A., Wojnicki, K., Wojtas, B., Gielniewski, B., Grajkowska, W., Kotulska, K., Szczylik, C., Czepko, R., Banach, M., et al. (2020). Aberrantly Expressed RECQL4 Helicase Supports Proliferation and Drug Resistance of Human Glioma Cells and Glioma Stem Cells. Cancers *12*, 2919. https://doi.org/10.3390/cancers12102919.

94. Gimple, R.C., Bhargava, S., Dixit, D., and Rich, J.N. (2019). Glioblastoma stem cells: lessons from the tumor hierarchy in a lethal cancer. Genes Dev. *33*, 591–609. https://doi.org/10.1101/gad.324301.119.

95. Prager, B.C., Bhargava, S., Mahadev, V., Hubert, C.G., and Rich, J.N. (2020). Glioblastoma Stem Cells: Driving Resilience through Chaos. Trends Cancer *6*, 223–235. https://doi.org/10.1016/j.trecan.2020.01.009.

96. Verhaak, R.G.W., Hoadley, K.A., Purdom, E., Wang, V., Qi, Y., Wilkerson, M.D., Miller, C.R., Ding, L., Golub, T., Mesirov, J.P., et al. (2010). Integrated Genomic Analysis Identifies Clinically Relevant Subtypes of Glioblastoma Characterized by Abnormalities in PDGFRA, IDH1, EGFR, and NF1. Cancer Cell *17*, 98–110. https://doi.org/10.1016/j.ccr.2009.12.020.

97. Brennan, C.W., Verhaak, R.G.W., McKenna, A., Campos, B., Noushmehr, H., Salama, S.R., Zheng, S., Chakravarty, D., Sanborn, J.Z., Berman, S.H., et al. (2013). The Somatic Genomic Landscape of Glioblastoma. Cell *155*, 462–477. https://doi.org/10.1016/j.cell.2013.09.034.

98. Frattini, V., Trifonov, V., Chan, J.M., Castano, A., Lia, M., Abate, F., Keir, S.T., Ji, A.X., Zoppoli, P., Niola, F., et al. (2013). The integrated landscape of driver genomic alterations in glioblastoma. Nat. Genet. *45*, 1141–1149. https://doi.org/10.1038/ng.2734.

99. Neftel, C., Laffy, J., Filbin, M.G., Hara, T., Shore, M.E., Rahme, G.J., Richman, A.R., Silverbush, D., Shaw, M.L., Hebert, C.M., et al. (2019). An Integrative Model of Cellular States, Plasticity, and Genetics for Glioblastoma. Cell *178*, 835–849.e21. https://doi.org/10.1016/j.cell.2019.06.024.

100. Wang, L.-B., Karpova, A., Gritsenko, M.A., Kyle, J.E., Cao, S., Li, Y., Rykunov, D., Colaprico, A., Rothstein, J.H., Hong, R., et al. (2021). Proteogenomic and metabolomic characterization of human glioblastoma. Cancer Cell *39*, 509–528.e20. https://doi.org/10.1016/j.ccell.2021.01.006.

101. McKinnon, C., Nandhabalan, M., Murray, S.A., and Plaha, P. (2021). Glioblastoma: clinical presentation, diagnosis, and management. BMJ *374*, n1560. https://doi.org/10.1136/bmj.n1560.

102. Ma, R., Taphoorn, M.J.B., and Plaha, P. (2021). Advances in the management of glioblastoma. J. Neurol. Neurosurg. Psychiatry *92*, 1103–1111. https://doi.org/10.1136/jnnp-2020-325334.

103. Wang, J., Cazzato, E., Ladewig, E., Frattini, V., Rosenbloom, D.I.S., Zairis, S., Abate, F., Liu, Z., Elliott, O., Shin, Y.-J., et al. (2016). Clonal evolution of glioblastoma under therapy. Nat. Genet. *48*, 768–776. https://doi.org/10.1038/ng.3590.

104. Muscat, A.M., Wong, N.C., Drummond, K.J., Algar, E.M., Khasraw, M., Verhaak, R., Field, K., Rosenthal, M.A., and Ashley, D.M. (2018). The evolutionary pattern of mutations in glioblastoma reveals therapy-mediated selection. Oncotarget *9*, 7844–7858. https://doi.org/10.18632/oncotarget.23541.

105. Zang, L., Kondengaden, S.M., Che, F., Wang, L., and Heng, X. (2018). Potential Epigenetic-Based Therapeutic Targets for Glioma. Front. Mol. Neurosci. *11*, 408.

106. Hutóczki, G., Virga, J., Birkó, Z., and Klekner, A. (2021). Novel Concepts of Glioblastoma Therapy Concerning Its Heterogeneity. Int. J. Mol. Sci. *22*, 10005. https://doi.org/10.3390/ijms221810005.

107. Gielniewski, B., Poleszak, K., Roura, A.-J., Szadkowska, P., Jacek, K., Krol, S.K., Guzik, R., Wiechecka, P., Maleszewska, M., Kaza, B., et al. (2023). Targeted sequencing of cancer-related genes reveals a recurrent TOP2A variant which affects DNA binding and coincides with global transcriptional changes in glioblastoma. Int. J. Cancer *153*, 1003–1015. https://doi.org/10.1002/ijc.34631.

108. Bhatti, G.K., Khullar, N., Sidhu, I.S., Navik, U.S., Reddy, A.P., Reddy, P.H., and Bhatti, J.S. (2021). Emerging role of non-coding RNA in health and disease. Metab. Brain Dis. *36*, 1119–1134. https://doi.org/10.1007/s11011-021-00739-y.

109. Shahzad, U., Krumholtz, S., Rutka, J.T., and Das, S. (2021). Noncoding RNAs in Glioblastoma: Emerging Biological Concepts and Potential Therapeutic Implications. Cancers *13*, 1555. https://doi.org/10.3390/cancers13071555.

110. Sharma, R.K., Calderon, C., and Vivas-Mejia, P.E. (2021). Targeting Non-coding RNA for Glioblastoma Therapy: The Challenge of Overcomes the Blood-Brain Barrier. Front. Med. Technol. *3*, 678593.

111. Mousavi, S.M., Derakhshan, M., Baharloii, F., Dashti, F., Mirazimi, S.M.A., Mahjoubin-Tehran, M., Hosseindoost, S., Goleij, P., Rahimian, N., Hamblin, M.R., and Mirzaei, H. (2022). Non-coding RNAs and glioblastoma: Insight into their roles in metastasis. Mol. Ther. Oncolytics *24*, 262–287. https://doi.org/10.1016/j.omto.2021.12.015.

112. Skalsky, R.L., and Cullen, B.R. (2011). Reduced Expression of Brain-Enriched microRNAs in Glioblastomas Permits Targeted Regulation of a Cell Death Gene. PLoS One *6*, e24248. https://doi.org/10.1371/journal.pone.0024248.

113. Papagiannakopoulos, T., Friedmann-Morvinski, D., Neveu, P., Dugas, J.C., Gill, R.M., Huillard, E., Liu, C., Zong, H., Rowitch, D.H., Barres, B.A., et al. (2012). Pro-neural miR-128 is a glioma tumor suppressor that targets mitogenic kinases. Oncogene *31*, 1884–1895. https://doi.org/10.1038/onc.2011.380.

114. Parsons, D.W., Jones, S., Zhang, X., Lin, J.C.-H., Leary, R.J., Angenendt, P., Mankoo, P., Carter, H., Siu, I.-M., Gallia, G.L., et al. (2008). An integrated genomic analysis of human glioblastoma multiforme. Science *321*, 1807–1812. https://doi.org/10.1126/science.1164382.

115. Maleszewska, M., and Kaminska, B. (2013). Is Glioblastoma an Epigenetic Malignancy? Cancers *5*, 1120–1139. https://doi.org/10.3390/cancers5031120.

116. Maleszewska, M., and Kaminska, B. (2015). Deregulation of histone-modifying enzymes and chromatin structure modifiers contributes to glioma development. Future Oncol. *11*, 2587–2601. https://doi.org/10.2217/fon.15.171.

117. Was, H., Krol, S.K., Rotili, D., Mai, A., Wojtas, B., Kaminska, B., and Maleszewska, M. (2019). Histone deacetylase inhibitors exert anti-tumor effects on human adherent and stem-like glioma cells. Clin. Epigenetics *11*, 11. https://doi.org/10.1186/s13148-018-0598-5.

118. Kaminska, B., Czapski, B., Guzik, R., Król, S.K., and Gielniewski, B. (2019). Consequences of IDH1/2 Mutations in Gliomas and an Assessment of Inhibitors Targeting Mutated IDH Proteins. Molecules *24*, 968. https://doi.org/10.3390/molecules24050968.

119. Stępniak, K., Machnicka, M.A., Mieczkowski, J., Macioszek, A., Wojtaś, B., Gielniewski, B., Poleszak, K., Perycz, M., Król, S.K., Guzik, R., et al. (2021). Mapping chromatin accessibility and active regulatory elements reveals pathological mechanisms in human gliomas. Nat. Commun. *12*, 3621. https://doi.org/10.1038/s41467-021-23922-2.

120. Bracken, A.P., and Helin, K. (2009). Polycomb group proteins: navigators of lineage pathways led astray in cancer. Nat. Rev. Cancer *9*, 773–784. https://doi.org/10.1038/nrc2736.

121. Pasini, D., and Di Croce, L. (2016). Emerging roles for Polycomb proteins in cancer. Curr. Opin. Genet. Dev. *36*, 50–58. https://doi.org/10.1016/j.gde.2016.03.013.

122. Pasini, D., Bracken, A.P., and Helin, K. (2004). Polycomb group proteins in cell cycle progression and cancer. Cell Cycle *3*, 396–400.

123. Sauvageau, M., and Sauvageau, G. (2010). Polycomb group proteins: multi-faceted regulators of somatic stem cells and cancer. Cell Stem Cell *7*, 299–313. https://doi.org/10.1016/j.stem.2010.08.002.

124. Richly, H., Aloia, L., and Di Croce, L. (2011). Roles of the Polycomb group proteins in stem cells and cancer. Cell Death Dis. *2*, e204. https://doi.org/10.1038/cddis.2011.84.

125. Bian, E.-B., Li, J., He, X.-J., Zong, G., Jiang, T., Li, J., and Zhao, B. (2014). Epigenetic modification in gliomas: role of the histone methyltransferase EZH2. Expert Opin. Ther. Targets *18*, 1197–1206. https://doi.org/10.1517/14728222.2014.941807.

126. Godlewski, J., Nowicki, M.O., Bronisz, A., Williams, S., Otsuki, A., Nuovo, G., Raychaudhury, A., Newton, H.B., Chiocca, E.A., and Lawler, S. (2008). Targeting of the Bmi-1 oncogene/stem cell renewal factor by microRNA-128 inhibits glioma proliferation and self-renewal. Cancer Res. *68*, 9125–9130. https://doi.org/10.1158/0008-5472.CAN-08-2629.

127. Shan, Z.N., Tian, R., Zhang, M., Gui, Z.H., Wu, J., Ding, M., Zhou, X.-F., and He, J. (2016). miR128-1 inhibits the growth of glioblastoma multiforme and glioma stem-like cells via targeting BMI1 and E2F3. Oncotarget *7*, 78813–78826. https://doi.org/10.18632/oncotarget.12385.

128. Häyry, V., Tanner, M., Blom, T., Tynninen, O., Roselli, A., Ollikainen, M., Sariola, H., Wartiovaara, K., and Nupponen, N.N. (2008). Copy number alterations of the polycomb gene BMI1 in gliomas. Acta Neuropathol. *116*, 97–102. https://doi.org/10.1007/s00401-008-0376-0.

129. Crea, F., Hurt, E.M., and Farrar, W.L. (2010). Clinical significance of Polycomb gene expression in brain tumors. Mol. Cancer *9*, 265. https://doi.org/10.1186/1476-4598-9-265.

130. Rooj, A.K., Ricklefs, F., Mineo, M., Nakano, I., Chiocca, E.A., Bronisz, A., and Godlewski, J. (2017). MicroRNA-Mediated Dynamic Bidirectional Shift between the Subclasses of Glioblastoma Stem-like Cells. Cell Rep. *19*, 2026–2032. https://doi.org/10.1016/j.celrep.2017.05.040.

131. Leeb, M., Pasini, D., Novatchkova, M., Jaritz, M., Helin, K., and Wutz, A. (2010). Polycomb complexes act redundantly to repress genomic repeats and genes. Genes Dev. *24*, 265–276. https://doi.org/10.1101/gad.544410.

132. Cohen, I., Bar, C., Liu, H., Valdes, V.J., Zhao, D., Galbo, P.M., Silva, J.M., Koseki, H., Zheng, D., and Ezhkova, E. (2021). Polycomb complexes redundantly maintain epidermal stem cell identity during development. Genes Dev. *35*, 354–366. https://doi.org/10.1101/gad.345363.120.

133. Zhu, Y., Dong, L., Wang, C., Hao, K., Wang, J., Zhao, L., Xu, L., Xia, Y., Jiang, Q., and Qin, J. (2022). Functional redundancy among Polycomb complexes in maintaining the pluripotent state of embryonic stem cells. Stem Cell Rep. *17*, 1198–1214. https://doi.org/10.1016/j.stemcr.2022.02.020.

134. Chou, D.M., Adamson, B., Dephoure, N.E., Tan, X., Nottke, A.C., Hurov, K.E., Gygi, S.P., Colaiácovo, M.P., and Elledge, S.J. (2010). A chromatin localization screen reveals poly (ADP ribose)-regulated recruitment of the repressive polycomb and NuRD complexes to sites of DNA damage. Proc. Natl. Acad. Sci. *107*, 18475–18480. https://doi.org/10.1073/pnas.1012946107.

135. Ismail, I.H., Andrin, C., McDonald, D., and Hendzel, M.J. (2010). BMI1-mediated histone ubiquitylation promotes DNA double-strand break repair. J. Cell Biol. *191*, 45–60. https://doi.org/10.1083/jcb.201003034.

136. Ginjala, V., Nacerddine, K., Kulkarni, A., Oza, J., Hill, S.J., Yao, M., Citterio, E., van Lohuizen, M., and Ganesan, S. (2011). BMI1 Is Recruited to DNA Breaks and Contributes to DNA Damage-Induced H2A Ubiquitination and Repair. Mol. Cell Biol. *31*, 1972–1982. https://doi.org/10.1128/MCB.00981-10.

137. Gieni, R.S., Ismail, I.H., Campbell, S., and Hendzel, M.J. (2011). Polycomb group proteins in the DNA damage response: A link between radiation resistance and "stemness.". Cell Cycle *10*, 883–894. https://doi.org/10.4161/cc.10.6.14907.

138. Liu, Z.-G., Liu, L., Xu, L.-H., Yi, W., Tao, Y.-L., Tu, Z.-W., Li, M.-Z., Zeng, M.-S., and Xia, Y.-F. (2012). Bmi-1 induces radioresistance in MCF-7 mammary carcinoma cells. Oncol. Rep. *27*, 1116–1122. https://doi.org/10.3892/or.2011.1615.

139. Facchino, S., Abdouh, M., Chatoo, W., and Bernier, G. (2010). BMI1 Confers Radioresistance to Normal and Cancerous Neural Stem Cells through Recruitment of the DNA Damage Response Machinery. J. Neurosci. *30*, 10096–10111. https://doi.org/10.1523/JNEUROSCI.1634-10.2010.

140. Li, J., Gong, L.-Y., Song, L.-B., Jiang, L.-L., Liu, L.-P., Wu, J., Yuan, J., Cai, J.-C., He, M., Wang, L., et al. (2010). Oncoprotein Bmi-1 Renders Apoptotic Resistance to Glioma Cells through Activation of the IKK-Nuclear Factor-κB Pathway. Am. J. Pathol. *176*, 699–709. https://doi.org/10.2353/ajpath.2010.090502.

141. Evangelisti, C., Florian, M.C., Massimi, I., Dominici, C., Giannini, G., Galardi, S., Buè, M.C., Massalini, S., McDowell, H.P., Messi, E., et al. (2009). MiR-128 up-regulation inhibits Reelin and DCX expression and reduces neuroblastoma cell motility and invasiveness. FASEB J *23*, 4276–4287. https://doi.org/10.1096/fj.09-134965.

142. Gao, Y., Zhang, R., Wei, G., Dai, S., Zhang, X., Yang, W., Li, X., and Bai, C. (2019). Long Non-coding RNA Maternally Expressed 3 Increases the Expression of Neuron-Specific Genes by Targeting miR-128-3p in All-Trans Retinoic Acid-Induced Neurogenic Differentiation From Amniotic Epithelial Cells. Front. Cell Dev. Biol. *7*, 342.

143. He, J., Liu, M.W., Wang, Z.Y., and Shi, R.J. (2022). Protective effects of the notoginsenoside R1 on acute lung injury by regulating the miR-128-2-5p/Tollip signaling pathway in rats with severe acute pancreatitis. Innate Immun. *28*, 19–36. https://doi.org/10.1177/17534259211068744.

144. Ghersi, J.J., Baldissera, G., Hintzen, J., Luff, S.A., Cheng, S., Xia, I.F., Sturgeon, C.M., and Nicoli, S. (2023). Haematopoietic stem and progenitor cell heterogeneity is inherited from the embryonic endothelium. Nat. Cell Biol. *25*, 1135–1145. https://doi.org/10.1038/s41556-023-01187-9.

145. Bhaskaran, V., Nowicki, M.O., Idriss, M., Jimenez, M.A., Lugli, G., Hayes, J.L., Mahmoud, A.B., Zane, R.E., Passaro, C., Ligon, K.L., et al. (2019). The functional synergism of microRNA clustering provides therapeutically relevant epigenetic interference in glioblastoma. Nat. Commun. *10*, 442. https://doi.org/10.1038/s41467-019-08390-z.

146. Salzman, J., Gawad, C., Wang, P.L., Lacayo, N., and Brown, P.O. (2012). Circular RNAs Are the Predominant Transcript Isoform from Hundreds of Human Genes in Diverse Cell Types. PLoS One *7*, e30733. https://doi.org/10.1371/journal.pone.0030733.

147. Hansen, T.B., Jensen, T.I., Clausen, B.H., Bramsen, J.B., Finsen, B., Damgaard, C.K., and Kjems, J. (2013). Natural RNA circles function as efficient microRNA sponges. Nature *495*, 384–388. https://doi.org/10.1038/nature11993.

148. Li, J.-H., Liu, S., Zhou, H., Qu, L.-H., and Yang, J.-H. (2014). starBase v2.0: decoding miRNA-ceRNA, miRNA-ncRNA and protein–RNA interaction networks from large-scale CLIP-Seq data. Nucleic Acids Res. *42*, D92–D97. https://doi.org/10.1093/nar/gkt1248.

149. Javanmard, A.-R., Jahanbakhshi, A., Nemati, H., Mowla, S.J., and Soltani, B.M. (2023). ADAMTS9-AS1 Long Non-coding RNA Sponges miR-128 and miR-150 to Regulate Ras/MAPK Signaling Pathway in Glioma. Cell. Mol. Neurobiol. *43*, 2309–2322. https://doi.org/10.1007/s10571-022-01311-7.

150. Zhao, H., Wang, Y., Liang, C., and Xie, M. (2023). LncRNA FOXD3-AS1/miR-128-3p axis-mediated IGF2BP3 in glioma stimulates cancer angiogenesis and progression. Folia Neuropathol. *61*, 168–184. https://doi.org/10.5114/fn.2023.126862.

151. Ye, Z., Guan, X., Shan, W., and Ma, B. (2023). OIP5-AS1 accelerates apoptosis of hippocampal neurons in cell models of epilepsy by modulating MiR-128-3p/BAX axis. Prev. Med. *173*, 107565. https://doi.org/10.1016/j.ypmed.2023.107565.

152. Hanan, M., Simchovitz, A., Yayon, N., Vaknine, S., Cohen-Fultheim, R., Karmon, M., Madrer, N., Rohrlich, T.M., Maman, M., Bennett, E.R., et al. (2020). A Parkinson's disease CircRNAs Resource reveals a link between circSLC8A1 and oxidative stress. EMBO Mol. Med. *12*, e11942. https://doi.org/10.15252/emmm.201911942.

153. Wang, C., Dong, M., Zhang, X., Wang, X., Zhao, Y., and Cao, Y. (2023). Competitive binding of circCCDC6 to microRNA-128-3p activates TXNIP/NLRP3 pathway and promotes cerebral ischemia-reperfusion defects. Acta Biochim. Pol. *70*, 807–815. https://doi.org/10.18388/abp.2020_6552.

154. Giza, D.E., Vasilescu, C., and Calin, G.A. (2014). MicroRNAs and ceRNAs: therapeutic implications of RNA networks. Expert Opin. Biol. Ther. *14*, 1285–1293. https://doi.org/10.1517/14712598.2014.920812.

155. Rinck, A., Preusse, M., Laggerbauer, B., Lickert, H., Engelhardt, S., and Theis, F.J. (2013). The human transcriptome is enriched for miRNA-binding sites located in cooperativity-permitting distance. RNA Biol. *10*, 1125–1135. https://doi.org/10.4161/rna.24955.

156. Mitra, R., Adams, C.M., Jiang, W., Greenawalt, E., and Eischen, C.M. (2020). Pan-cancer analysis reveals cooperativity of both strands of microRNA that regulate tumorigenesis and patient survival. Nat. Commun. *11*, 968. https://doi.org/10.1038/s41467-020-14713-2.

157. Thomas, K.T., Gross, C., and Bassell, G.J. (2018). microRNAs Sculpt Neuronal Communication in a Tight Balance That Is Lost in Neurological Disease. Front. Mol. Neurosci. *11*, 455.

158. Santos, M.C.T., Tegge, A.N., Correa, B.R., Mahesula, S., Kohnke, L.Q., Qiao, M., Ferreira, M.A.R., Kokovay, E., and Penalva, L.O.F. (2016). miR-124, -128, and -137 Orchestrate Neural Differentiation by Acting on Overlapping Gene Sets Containing a Highly Connected Transcription Factor Network. Stem Cell. *34*, 220–232. https://doi.org/10.1002/stem.2204.

159. Wang, Y., Chen, R., Zhou, X., Guo, R., Yin, J., Li, Y., and Ma, G. (2020). miR-137: A Novel Therapeutic Target for Human Glioma. Mol. Ther. Nucleic Acids *21*, 614–622. https://doi.org/10.1016/j.omtn.2020.06.028.

160. Lee, S.W., Oh, Y.M., Lu, Y.-L., Kim, W.K., and Yoo, A.S. (2018). MicroRNAs Overcome Cell Fate Barrier by Reducing EZH2-Controlled REST Stability during Neuronal Conversion of Human Adult Fibroblasts. Dev. Cell *46*, 73–84.e7. https://doi.org/10.1016/j.devcel.2018.06.007.

161. Kennerdell, J.R., Liu, N., and Bonini, N.M. (2018). MiR-34 inhibits polycomb repressive complex 2 to modulate chaperone expression and promote healthy brain aging. Nat. Commun. *9*, 4188. https://doi.org/10.1038/s41467-018-06592-5.

162. Li, S., Wei, X., He, J., Cao, Q., Du, D., Zhan, X., Zeng, Y., Yuan, S., and Sun, L. (2021). The comprehensive landscape of miR-34a in cancer research. Cancer Metastasis Rev. *40*, 925–948. https://doi.org/10.1007/s10555-021-09973-3.

163. Poddar, S., Kesharwani, D., and Datta, M. (2017). Interplay between the miRNome and the epigenetic machinery: Implications in health and disease. J. Cell. Physiol. *232*, 2938–2945. https://doi.org/10.1002/jcp.25819.

164. O'Loghlen, A., Brookes, S., Martin, N., Rapisarda, V., Peters, G., and Gil, J. (2015). CBX7 and miR-9 are part of an autoregulatory loop controlling p16INK4a. Aging Cell *14*, 1113–1121. https://doi.org/10.1111/acel.12404.

165. Huang, W., Feng, Y., Liang, J., Yu, H., Wang, C., Wang, B., Wang, M., Jiang, L., Meng, W., Cai, W., et al. (2018). Loss of microRNA-128 promotes cardiomyocyte proliferation and heart regeneration. Nat. Commun. *9*, 700. https://doi.org/10.1038/s41467-018-03019-z.

166. Geng, Y.-B., Pan, C.-C., Xu, C., Zuo, P.-C., Wang, Y., Li, X.-O., and Zhang, L.-W. (2020). Long non-coding RNA LINC00346 regulates proliferation and apoptosis by targeting miR-128-3p/SZRD1 axis in glioma. Eur. Rev. Med. Pharmacol. Sci. *24*, 9581–9590. https://doi.org/10.26355/eurrev_202009_23046.

167. Jalali, S., Singh, A., Maiti, S., and Scaria, V. (2017). Genome-wide computational analysis of potential long noncoding RNA mediated DNA:DNA:RNA triplexes in the human genome. J. Transl. Med. *15*, 186. https://doi.org/10.1186/s12967-017-1282-9.

168. Ma, Y., Wang, P., Xue, Y., Qu, C., Zheng, J., Liu, X., Ma, J., and Liu, Y. (2017). PVT1 affects growth of glioma microvascular endothelial cells by negatively regulating miR-186. Tumor Biol. *39*. 1010428317694326. https://doi.org/10.1177/1010428317694326.

169. Fu, C., Li, D., Zhang, X., Liu, N., Chi, G., and Jin, X. (2018). LncRNA PVT1 Facilitates Tumorigenesis and Progression of Glioma via Regulation of MiR-128-3p/GREM1 Axis and BMP Signaling Pathway. Neurotherapeutics *15*, 1139–1157. https://doi.org/10.1007/s13311-018-0649-9.

170. Dahai, Z., Daliang, C., Famu, L., Xiang, W., Lenian, L., Jianmin, C., and Xiaobing, X. (2020). Lowly expressed lncRNA PVT1 suppresses proliferation and advances apoptosis of glioma cells through up-regulating microRNA-128-1-5p and inhibiting PTBP1. Brain Res. Bull. *163*, 1–13. https://doi.org/10.1016/j.brainresbull.2020.06.006.

171. Wang, C., Yu, G., Xu, Y., Liu, C., Sun, Q., Li, W., Sun, J., Jiang, Y., and Ye, L. (2021). Knockdown of Long Non-Coding RNA HCP5 Increases Radiosensitivity Through Cellular Senescence by Regulating microRNA-128 in Gliomas. Cancer Manag. Res. *13*, 3723–3737. https://doi.org/10.2147/CMAR.S301333.

172. Chen, J., Wang, H., Wang, J., Niu, W., Deng, C., and Zhou, M. (2021). LncRNA NEAT1 Enhances Glioma Progression via Regulating the miR-128-3p/ITGA5 Axis. Mol. Neurobiol. *58*, 5163–5177. https://doi.org/10.1007/s12035-021-02474-y.

173. Xian, S., Ding, R., Li, M., and Chen, F. (2021). LncRNA NEAT1/miR-128-3p/AQP4 axis regulating spinal cord injury-induced neuropathic pain progression. J. Neuroimmunol. *351*, 577457. https://doi.org/10.1016/j.jneuroim.2020.577457.

174. Jiang, Y., Wang, Y., Sun, Y., and Jiang, H. (2020). Long non-coding RNA Peg13 attenuates the sevoflurane toxicity against neural stem cells by sponging microRNA-128-3p to preserve Sox13 expression. PLoS One *15*, e0243644. https://doi.org/10.1371/journal.pone.0243644.

175. Wang, L., Zhang, Z., and Wang, H. (2021). Downregulation of lncRNA GAS5 prevents mitochondrial apoptosis and hypoxic-ischemic brain damage in neonatal rats through the microRNA-128-3p/Bax/Akt/GSK-3β axis. Neuroreport *32*, 1395–1402. https://doi.org/10.1097/WNR.0000000000001730.

176. Liang, X., Shangguan, W., Zhang, M., Mei, S., Wang, L., and Yang, R. (2017). miR-128 enhances dendritic cell-mediated anti-tumor immunity via targeting of p38. Mol. Med. Rep. *16*, 1307–1313. https://doi.org/10.3892/mmr.2017.6717.

177. Xi, Q., Chen, Y., Yang, G.-Z., Zhang, J.-Y., Zhang, L.-J., Guo, X.-D., Zhao, J.-Y., Xue, Z.-Y., Li, Y., and Zhang, R. (2020). miR-128 Regulates Tumor Cell CD47 Expression and Promotes Anti-tumor Immunity in Pancreatic Cancer. Front. Immunol. *11*, 890.

178. Leclair, P., and Lim, C.J. (2020). CD47 (Cluster of differentiation 47): an anti-phagocytic receptor with a multitude of signaling functions. Anim. Cells Syst. *24*, 243–252. https://doi.org/10.1080/19768354.2020.1818618.

179. Moffett, H.F. (2012). MicroRNAs in Normal and Malignant Lymphocytes.

180. Reardon, D.A., Gokhale, P.C., Klein, S.R., Ligon, K.L., Rodig, S.J., Ramkissoon, S.H., Jones, K.L., Conway, A.S., Liao, X., Zhou, J., et al. (2016). Glioblastoma Eradication Following Immune Checkpoint Blockade in an Orthotopic, Immunocompetent Model. Cancer Immunol. Res. *4*, 124–135. https://doi.org/10.1158/2326-6066.CIR-15-0151.

181. Caccese, M., Indraccolo, S., Zagonel, V., and Lombardi, G. (2019). PD-1/PD-L1 immune-checkpoint inhibitors in glioblastoma: A concise review. Crit. Rev. Oncol. Hematol. *135*, 128–134. https://doi.org/10.1016/j.critrevonc.2018.12.002.

182. Yang, T., Kong, Z., and Ma, W. (2021). PD-1/PD-L1 immune checkpoint inhibitors in glioblastoma: clinical studies, challenges and potential. Hum. Vaccin. Immunother. *17*, 546–553. https://doi.org/10.1080/21645515.2020.1782692.

183. Chan, H.Y., Choi, J., Jackson, C., and Lim, M. (2021). Combination immunotherapy strategies for glioblastoma. J. Neuro Oncol. *151*, 375–391. https://doi.org/10.1007/s11060-020-03481-0.

184. Khorkova, O., and Wahlestedt, C. (2017). Oligonucleotide therapies for disorders of the nervous system. Nat. Biotechnol. *35*, 249–263. https://doi.org/10.1038/nbt.3784.

185. Levin, A.A. (2019). Treating Disease at the RNA Level with Oligonucleotides. N. Engl. J. Med. *380*, 57–70. https://doi.org/10.1056/NEJMra1705346.

186. Hoy, S.M. (2021). Nusinersen: A Review in 5q Spinal Muscular Atrophy. CNS Drugs *35*, 1317–1328. https://doi.org/10.1007/s40263-021-00878-x.

187. Gidaro, T., and Servais, L. (2019). Nusinersen treatment of spinal muscular atrophy: current knowledge and existing gaps. Dev. Med. Child Neurol. *61*, 19–24. https://doi.org/10.1111/dmcn.14027.

188. Zhang, Y., Wang, Z., and Gemeinhart, R.A. (2013). Progress in microRNA delivery. J. Control. Release *172*, 962–974. https://doi.org/10.1016/j.jconrel.2013.09.015.

189. Sun, P., Liu, D.Z., Jickling, G.C., Sharp, F.R., and Yin, K.-J. (2018). MicroRNA-based therapeutics in central nervous system injuries. J. Cereb. Blood Flow Metab. *38*, 1125–1148. https://doi.org/10.1177/0271678X18773871.

190. Hu, B., Zhong, L., Weng, Y., Peng, L., Huang, Y., Zhao, Y., and Liang, X.-J. (2020). Therapeutic siRNA: state of the art. Signal Transduct. Target. Ther. *5*, 101–125. https://doi.org/10.1038/s41392-020-0207-x.

191. Broderick, J.A., and Zamore, P.D. (2011). MicroRNA therapeutics. Gene Ther. *18*, 1104–1110. https://doi.org/10.1038/gt.2011.50.

192. Lanza, M., Cuzzocrea, S., Oddo, S., Esposito, E., and Casili, G. (2023). The Role of miR-128 in Neurodegenerative Diseases. Int. J. Mol. Sci. *24*, 6024. https://doi.org/10.3390/ijms24076024.

193. Decressac, M., Mattsson, B., Weikop, P., Lundblad, M., Jakobsson, J., and Björklund, A. (2013). TFEB-mediated autophagy rescues midbrain dopamine neurons from α-synuclein toxicity. Proc. Natl. Acad. Sci. *110*, E1817–E1826. https://doi.org/10.1073/pnas.1305623110.

194. Yaida, Y., and Nowak, T.S. (1995). Distribution of phosphodiester and phosphorothioate oligonucleotides in rat brain after intraventricular and intrahippocampal administration determined by in situ hybridization. Regul. Pept. *59*, 193–199. https://doi.org/10.1016/0167-0115(95)00100.

195. Rigo, F., Chun, S.J., Norris, D.A., Hung, G., Lee, S., Matson, J., Fey, R.A., Gaus, H., Hua, Y., Grundy, J.S., et al. (2014). Pharmacology of a Central Nervous System Delivered 2′-O-Methoxyethyl–Modified Survival of Motor Neuron Splicing Oligonucleotide in Mice and Nonhuman Primates. J. Pharmacol. Exp. Ther. *350*, 46–55. https://doi.org/10.1124/jpet.113.212407.

196. Dean, D.A. (2000). Peptide nucleic acids: versatile tools for gene therapy strategies. Adv. Drug Deliv. Rev. *44*, 81–95. https://doi.org/10.1016/S0169-409X(00)00087-9.

197. Wahlestedt, C., Salmi, P., Good, L., Kela, J., Johnsson, T., Hökfelt, T., Broberger, C., Porreca, F., Lai, J., Ren, K., et al. (2000). Potent and nontoxic antisense oligonucleotides containing locked nucleic acids. Proc. Natl. Acad. Sci. *97*, 5633–5638. https://doi.org/10.1073/pnas.97.10.5633.

198. Davis, S., Lollo, B., Freier, S., and Esau, C. (2006). Improved targeting of miRNA with antisense oligonucleotides. Nucleic Acids Res. *34*, 2294–2304. https://doi.org/10.1093/nar/gkl183.

199. Oh, S.Y., Ju, Y., Kim, S., and Park, H. (2010). PNA-Based Antisense Oligonucleotides for MicroRNAs Inhibition in the Absence of a Transfection Reagent. Oligonucleotides *20*, 225–230. https://doi.org/10.1089/oli.2010.0238.

200. Liang, H., Jiao, Z., Rong, W., Qu, S., Liao, Z., Sun, X., Wei, Y., Zhao, Q., Wang, J., Liu, Y., et al. (2020). 3′-Terminal 2′-O-methylation of lung cancer miR-21-5p enhances its stability and association with Argonaute 2. Nucleic Acids Res. *48*, 7027–7040. https://doi.org/10.1093/nar/gkaa504.

201. Chen, P.Y., Weinmann, L., Gaidatzis, D., Pei, Y., Zavolan, M., Tuschl, T., and Meister, G. (2008). Strand-specific 5′-O-methylation of siRNA duplexes controls guide strand selection and targeting specificity. RNA *14*, 263–274. https://doi.org/10.1261/rna.789808.

202. van Rooij, E., Purcell, A.L., and Levin, A.A. (2012). Developing MicroRNA Therapeutics. Circ. Res. *110*, 496–507. https://doi.org/10.1161/CIRCRESAHA.111.247916.

203. Teixeira, M.I., Lopes, C.M., Amaral, M.H., and Costa, P.C. (2023). Surface-modified lipid nanocarriers for crossing the blood-brain barrier (BBB): A current overview of active targeting in brain diseases. Colloids Surf. B Biointerfaces *221*, 112999. https://doi.org/10.1016/j.colsurfb.2022.112999.

204. Priya, S., Desai, V.M., and Singhvi, G. (2023). Surface Modification of Lipid-Based Nanocarriers: A Potential Approach to Enhance Targeted Drug Delivery. ACS Omega *8*, 74–86. https://doi.org/10.1021/acsomega.2c05976.

205. Nguyen, L.D., Wei, Z., Silva, M.C., Barberán-Soler, S., Zhang, J., Rabinovsky, R., Muratore, C.R., Stricker, J.M.S., Hortman, C., Young-Pearse, T.L., et al. (2023). Small molecule regulators of microRNAs identified by high-throughput screen coupled with high-throughput sequencing. Nat. Commun. *14*, 7575. https://doi.org/10.1038/s41467-023-43293-0.

206. Chen, M., Medarova, Z., and Moore, A. (2021). Role of microRNAs in glioblastoma. Oncotarget *12*, 1707–1723. https://doi.org/10.18632/oncotarget.28039.

207. Mafi, A., Rahmati, A., Babaei Aghdam, Z., Salami, R., Salami, M., Vakili, O., and Aghadavod, E. (2022). Recent insights into the microRNA-dependent modulation of gliomas from pathogenesis to diagnosis and treatment. Cell. Mol. Biol. Lett. *27*, 65. https://doi.org/10.1186/s11658-022-00354-4.

208. Christopher, A.F., Kaur, R.P., Kaur, G., Kaur, A., Gupta, V., and Bansal, P. (2016). MicroRNA therapeutics: Discovering novel targets and developing specific therapy. Perspect. Clin. Res. *7*, 68–74. https://doi.org/10.4103/2229-3485.179431.

209. Glinge, C., Clauss, S., Boddum, K., Jabbari, R., Jabbari, J., Risgaard, B., Tomsits, P., Hildebrand, B., Kääb, S., Wakili, R., et al. (2017). Stability of Circulating

Blood-Based MicroRNAs - Pre-Analytic Methodological Considerations. PLoS One *12*, e0167969. https://doi.org/10.1371/journal.pone.0167969.

210. Sempere, L.F., Freemantle, S., Pitha-Rowe, I., Moss, E., Dmitrovsky, E., and Ambros, V. (2004). Expression profiling of mammalian microRNAs uncovers a subset of brain-expressed microRNAs with possible roles in murine and human neuronal differentiation. Genome Biol. *5*, R13. https://doi.org/10.1186/gb-2004-5-3-r13.

211. Bronisz, A., Wang, Y., Nowicki, M.O., Peruzzi, P., Ansari, K., Ogawa, D., Balaj, L., De Rienzo, G., Mineo, M., Nakano, I., et al. (2014). Extracellular Vesicles Modulate the Glioblastoma Microenvironment via a Tumor Suppression Signaling Network Directed by miR-1. Cancer Res. *74*, 738–750. https://doi.org/10.1158/0008-5472.CAN-13-2650.

212. Palazzolo, S., Memeo, L., Hadla, M., Duzagac, F., Steffan, A., Perin, T., Canzonieri, V., Tuccinardi, T., Caligiuri, I., and Rizzolio, F. (2020). Cancer Extracellular Vesicles: Next-Generation Diagnostic and Drug Delivery Nanotools. Cancers *12*, 3165. https://doi.org/10.3390/cancers12113165.

213. Wiklander, O.P.B., Nordin, J.Z., O'Loughlin, A., Gustafsson, Y., Corso, G., Mäger, I., Vader, P., Lee, Y., Sork, H., Seow, Y., et al. (2015). Extracellular vesicle in vivo biodistribution is determined by cell source, route of administration and targeting. J. Extracell. Vesicles *4*, 26316. https://doi.org/10.3402/jev.v4.26316.

214. Batrakova, E.V., and Kim, M.S. (2015). Using exosomes, naturally-equipped nanocarriers, for drug delivery. J. Control. Release *219*, 396–405. https://doi.org/10.1016/j.jconrel.2015.07.030.

215. Zhao, C., Busch, D.J., Vershel, C.P., and Stachowiak, J.C. (2016). Multifunctional Transmembrane Protein Ligands for Cell-Specific Targeting of Plasma Membrane-Derived Vesicles. Small *12*, 3837–3848. https://doi.org/10.1002/smll.201600493.

216. Sil, S., Dagur, R.S., Liao, K., Peeples, E.S., Hu, G., Periyasamy, P., and Buch, S. (2020). Strategies for the use of Extracellular Vesicles for the Delivery of Therapeutics. J. Neuroimmune Pharmacol. *15*, 422–442. https://doi.org/10.1007/s11481-019-09873-y.

217. Bellavia, D., Raimondo, S., Calabrese, G., Forte, S., Cristaldi, M., Patinella, A., Memeo, L., Manno, M., Raccosta, S., Diana, P., et al. (2017). Interleukin 3- receptor targeted exosomes inhibit *in vitro* and *in vivo* Chronic Myelogenous Leukemia cell growth. Theranostics *7*, 1333–1345. https://doi.org/10.7150/thno.17092.

218. Wang, Y., Chen, X., Tian, B., Liu, J., Yang, L., Zeng, L., Chen, T., Hong, A., and Wang, X. (2017). Nucleolin-targeted Extracellular Vesicles as a Versatile Platform for Biologics Delivery to Breast Cancer. Theranostics *7*, 1360–1372. https://doi.org/10.7150/thno.16532.

219. Alvarez-Erviti, L., Seow, Y., Yin, H., Betts, C., Lakhal, S., and Wood, M.J.A. (2011). Delivery of siRNA to the mouse brain by systemic injection of targeted exosomes. Nat. Biotechnol. *29*, 341–345. https://doi.org/10.1038/nbt.1807.

220. Yuyama, K., Sun, H., Sakai, S., Mitsutake, S., Okada, M., Tahara, H., Furukawa, J.I., Fujitani, N., Shinohara, Y., and Igarashi, Y. (2014). Decreased Amyloid-β Pathologies by Intracerebral Loading of Glycosphingolipid-enriched Exosomes in Alzheimer Model Mice. J. Biol. Chem. *289*, 24488–24498. https://doi.org/10.1074/jbc.M114.577213.

221. Godlewski, J., Ferrer-Luna, R., Rooj, A.K., Mineo, M., Ricklefs, F., Takeda, Y.S., Nowicki, M.O., Salińska, E., Nakano, I., Lee, H., et al. (2017). MicroRNA Signatures and Molecular Subtypes of Glioblastoma: The Role of Extracellular Transfer. Stem Cell Rep. *8*, 1497–1505. https://doi.org/10.1016/j.stemcr.2017.04.024.

222. Kowal, J., Arras, G., Colombo, M., Jouve, M., Morath, J.P., Primdal-Bengtson, B., Dingli, F., Loew, D., Tkach, M., and Théry, C. (2016). Proteomic comparison defines novel markers to characterize heterogeneous populations of extracellular vesicle subtypes. Proc. Natl. Acad. Sci. *113*, E968–E977. https://doi.org/10.1073/pnas.1521230113.

223. Armstrong, J.P.K., and Stevens, M.M. (2018). Strategic design of extracellular vesicle drug delivery systems. Adv. Drug Deliv. Rev. *130*, 12–16. https://doi.org/10.1016/j.addr.2018.06.017.

224. Bovy, N., Blomme, B., Frères, P., Dederen, S., Nivelles, O., Lion, M., Carnet, O., Martial, J.A., Noël, A., Thiry, M., et al. (2015). Endothelial exosomes contribute to the antitumor response during breast cancer neoadjuvant chemotherapy via microRNA transfer. Oncotarget 6, 10253–10266.

225. Hannafon, B.N., Carpenter, K.J., Berry, W.L., Janknecht, R., Dooley, W.C., and Ding, W.-Q. (2015). Exosome-mediated microRNA signaling from breast cancer cells is altered by the anti-angiogenesis agent docosahexaenoic acid (DHA). Mol. Cancer *14*, 133. https://doi.org/10.1186/s12943-015-0400-7.

226. Xiao, F., zhang, W., Chen, L., Chen, F., Xie, H., Xing, C., Yu, X., Ding, S., Chen, K., Guo, H., et al. (2013). MicroRNA-503 inhibits the G1/S transition by downregulating cyclin D3 and E2F3 in hepatocellular carcinoma. J. Transl. Med. *11*, 195. https://doi.org/10.1186/1479-5876-11-195.

227. Lou, G., Song, X., Yang, F., Wu, S., Wang, J., Chen, Z., and Liu, Y. (2015). Exosomes derived from miR-122-modified adipose tissue-derived MSCs increase chemosensitivity of hepatocellular carcinoma. J. Hematol. Oncol. *8*, 122. https://doi.org/10.1186/s13045-015-0220-7.

228. Qiu, T., Zhou, L., Wang, T., Xu, J., Wang, J., Chen, W., Zhou, X., Huang, Z., Zhu, W., Shu, Y., and Liu, P. (2013). miR-503 regulates the resistance of non-small cell lung cancer cells to cisplatin by targeting Bcl-2. Int. J. Mol. Med. *32*, 593–598. https://doi.org/10.3892/ijmm.2013.1439.

229. Yang, Y., Liu, L., Zhang, Y., Guan, H., Wu, J., Zhu, X., Yuan, J., and Li, M. (2014). MiR-503 targets PI3K p85 and IKK-β and suppresses progression of non-small cell lung cancer. Int. J. Cancer *135*, 1531–1542. https://doi.org/10.1002/ijc.28799.

230. Vázquez-Ríos, A.J., Molina-Crespo, Á., Bouzo, B.L., López-López, R., Moreno-Bueno, G., and de la Fuente, M. (2019). Exosome-mimetic nanoplatforms for targeted cancer drug delivery. J. Nanobiotechnology *17*, 85. https://doi.org/10.1186/s12951-019-0517-8.

231. Akinc, A., Zumbuehl, A., Goldberg, M., Leshchiner, E.S., Busini, V., Hossain, N., Bacallado, S.A., Nguyen, D.N., Fuller, J., Alvarez, R., et al. (2008). A combinatorial library of lipid-like materials for delivery of RNAi therapeutics. Nat. Biotechnol. *26*, 561–569. https://doi.org/10.1038/nbt1402.

232. Akinc, A., Goldberg, M., Qin, J., Dorkin, J.R., Gamba-Vitalo, C., Maier, M., Jayaprakash, K.N., Jayaraman, M., Rajeev, K.G., Manoharan, M., et al. (2009). Development of lipidoid-siRNA formulations for systemic delivery to the liver. Mol. Ther. *17*, 872–879. https://doi.org/10.1038/mt.2009.36.

233. Dong, Y., Love, K.T., Dorkin, J.R., Sirirungruang, S., Zhang, Y., Chen, D., Bogorad, R.L., Yin, H., Chen, Y., Vegas, A.J., et al. (2014). Lipopeptide nanoparticles for potent and selective siRNA delivery in rodents and nonhuman primates. Proc. Natl. Acad. Sci. *111*, 3955–3960. https://doi.org/10.1073/pnas.1322937111.

234. Kauffman, K.J., Dorkin, J.R., Yang, J.H., Heartlein, M.W., DeRosa, F., Mir, F.F., Fenton, O.S., and Anderson, D.G. (2015). Optimization of Lipid Nanoparticle Formulations for mRNA Delivery in Vivo with Fractional Factorial and Definitive Screening Designs. Nano Lett. *15*, 7300–7306. https://doi.org/10.1021/acs.nanolett.5b02497.

235. Fenton, O.S., Kauffman, K.J., McClellan, R.L., Appel, E.A., Dorkin, J.R., Tibbitt, M.W., Heartlein, M.W., DeRosa, F., Langer, R., and Anderson, D.G. (2016). Bioinspired Alkenyl Amino Alcohol Ionizable Lipid Materials for Highly Potent In Vivo mRNA Delivery. Adv. Mater. *28*, 2939–2943. https://doi.org/10.1002/adma.201505822.

236. Kaczmarek, J.C., Kowalski, P.S., and Anderson, D.G. (2017). Advances in the delivery of RNA therapeutics: from concept to clinical reality. Genome Med. *9*, 60. https://doi.org/10.1186/s13073-017-0450-0.

OMTN, Volume *35*

Supplemental information

MiR-128-3p – a gray eminence of the human central nervous system

Klaudia Kiel, Sylwia Katarzyna Król, Agnieszka Bronisz, and Jakub Godlewski

SUPPLEMENTAL MATERIALS AND METHODS

Databases and Data Selection

For the examination of miR-128 gene family expression in the human body and brain compartments, the RNA Tissue Atlas dataset was employed [1]. Briefly, data in the miRNATissueAtlas2 were generated using a stand-alone version of our web-based tool miRMaster [2, 3]. The most recent online version supports the same features as the stand-alone version, most notably the multi-species support [4]. We conducted all analyses using the standard parameter settings as in the online version. The following ncRNA databases are included in the analysis: miRBase version 22.1 [5], Ensembl ncRNA version 100 [6], RNACentral version 15 [7], GtRNAdb version 18.1 [8] and NONCODE version 5 [9]. Subsequently, miEAA - microRNA Enrichment and Annotation Analysis [10] results were incorporated for review, utilizing the Gene Ontology (GO) database (December 2023 edition) and Shine Go version 0.79 of the application [11].

The source code is accessible at https://github.com/iDEP-SDSU/idep/tree/master/shinyapps/go61. Current database files are available at https://doi.org/10.5281/zenodo.1451847.
All data included in the miRNATissueAtlas2 are freely accessible from the Gene Expression Omnibus (GSE163534).

MicroRNA Expression Analysis

Expression Mean, log-transformed, normalized values (>0.01 RKPM) from the RNA Tissue Atlas databases were utilized to define microRNA signatures based on the expression of mature transcripts from whole brain tissue and its compartments. Data collection adheres to all relevant laws, regulations, and policies for protecting human subjects (see Table S1).

Functional and Statistical Analysis

A hierarchical clustering tree summarizes the correlation among significant Pathways databases listed in the Figure Legend [12-18] for miR-128-3p target genes input (n=1225) selected based on Target Scan v8 [16]. All statistical operations were performed with GraphPad Prism 8 software/ excel, considering significance with a False Discovery Rate (FDR > 0.05) with corrected q-value < 0.01 and correlation r value = +/-0.5.

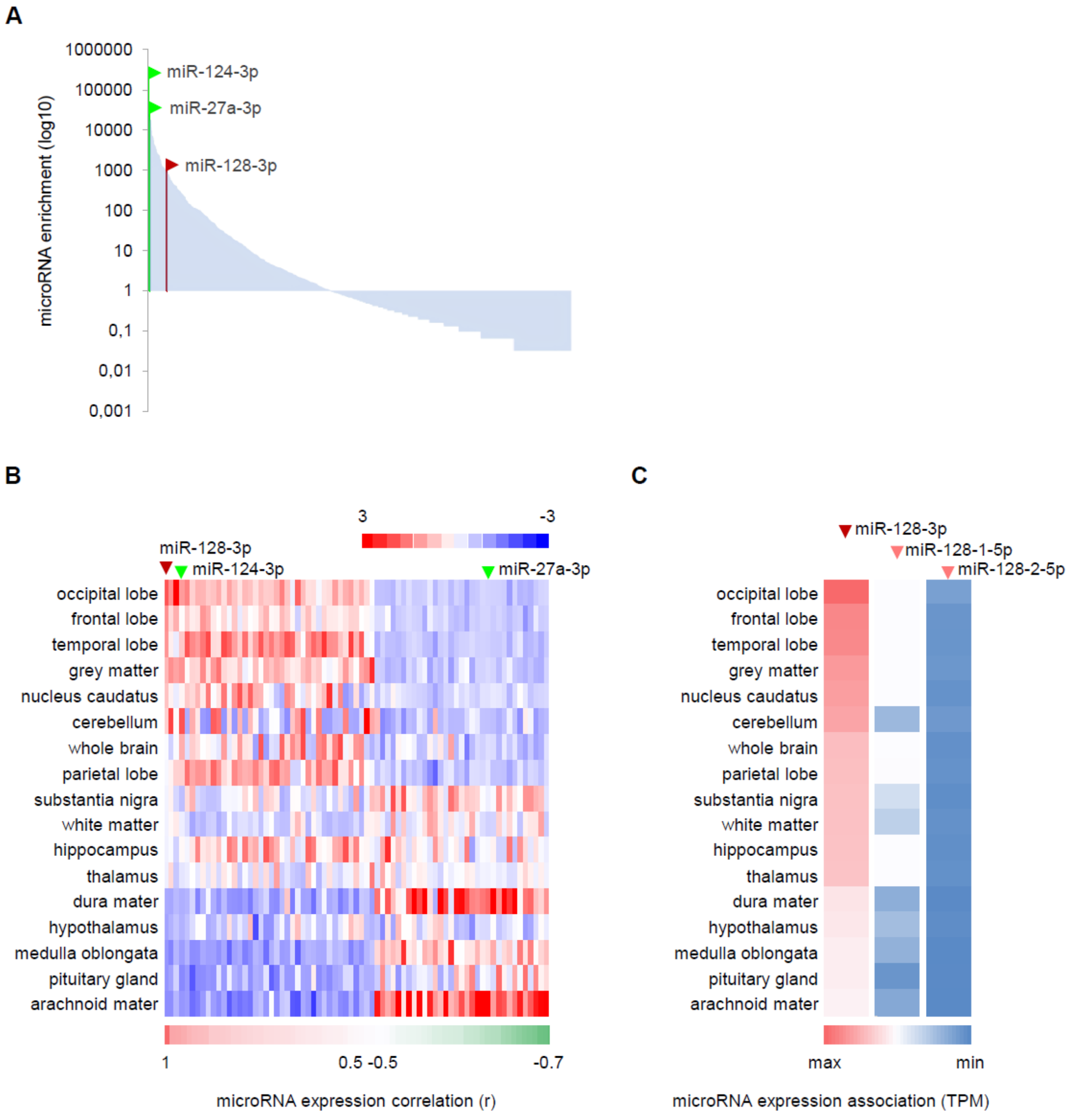

Figure S1. Brain tissue microRNAome overview.
A MicroRNAome enrichment in brain tissue is based on mean, log-transformed, normalized values (>0.01) downloaded from the RNA Tissue Atlas database (Table S1/Spreadsheet: 2 and 3). Mature microRNA n=1595.

B MiR-128-3p microRNAome expression correlation brain tissue compartments matrix is based on mean, log-transformed, normalized values (value > 0.01), downloaded from the RNA Tissue Atlas database and correlated with miR-128-3p with r value ≥ ±0.5. (Table S1/Spreadsheet: 4). Mature microRNA n=73.

C MiR-128-3p family mature microRNAs expression association heat map is based on mean, log-transformed, normalized values of miR-128-3p transcripts downloaded from the RNA Tissue Atlas database (Table S1/Spreadsheet: 5). Mature microRNA n=3. TPM - transcripts per kilobase million.

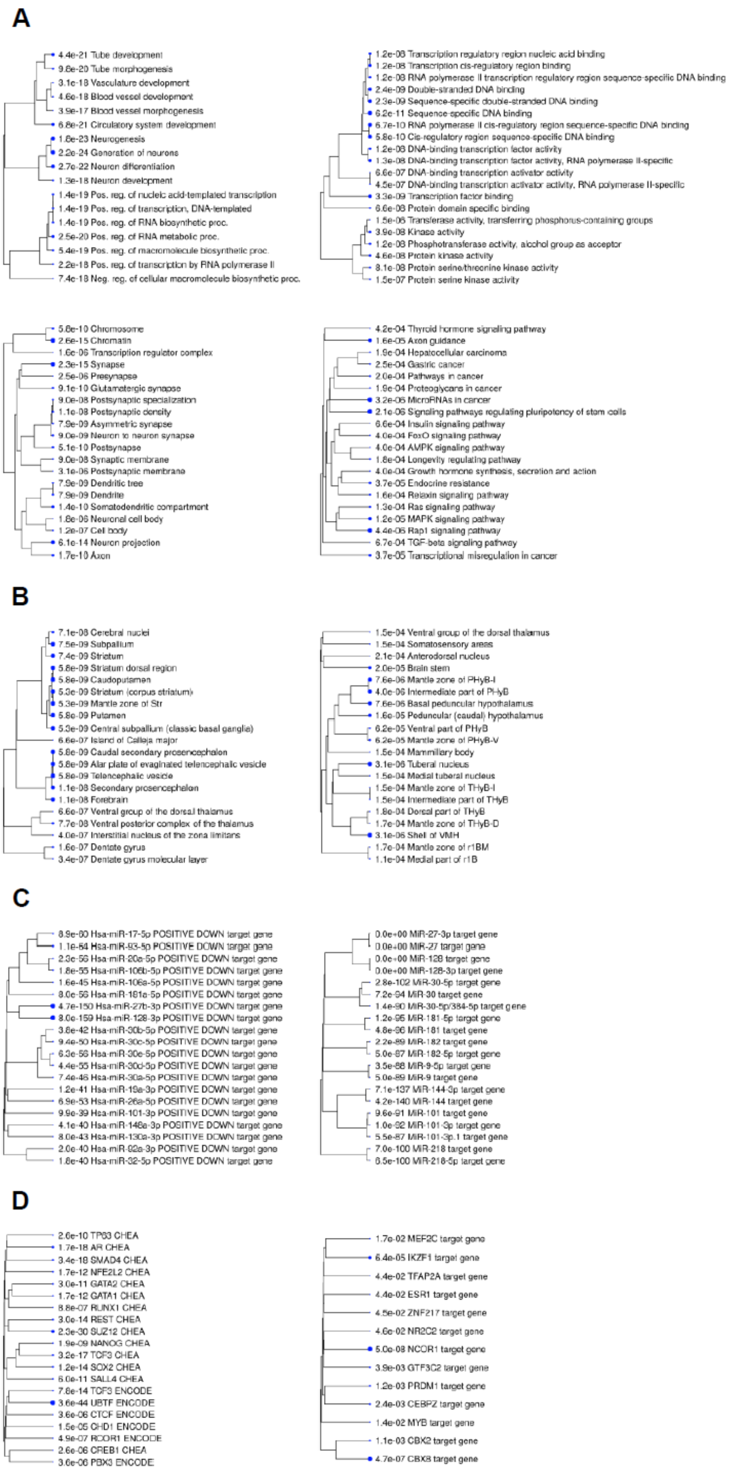
A
4.4e-21 Tube development
9.8e-20 Tube morphogenesis
3.1e-18 Vasculature development
4.6e-18 Blood vessel development
3.9e-17 Blood vessel morphogenesis
6.8e-21 Circulatory system development
1.8e-23 Neurogenesis
2.2e-24 Generation of neurons
2.7e-22 Neuron differentiation
1.3e-18 Neuron development
1.4e-19 Pos. reg. of nucleic acid-templated transcription
1.4e-19 Pos. reg. of transcription, DNA-templated
1.4e-19 Pos. reg. of RNA biosynthetic proc.
2.5e-20 Pos. reg. of RNA metabolic proc.
5.4e-19 Pos. reg. of macromolecule biosynthetic proc.
2.2e-18 Pos. reg. of transcription by RNA polymerase II
7.4e-18 Neg. reg. of cellular macromolecule biosynthetic proc.
1.2e-08 Transcription regulatory region nucleic acid binding
1.2e-08 Transcription cis-regulatory region binding
1.2e-08 RNA polymerase II transcription regulatory region sequence-specific DNA binding
2.4e-09 Double-stranded DNA binding
2.3e-09 Sequence-specific double-stranded DNA binding
6.2e-11 Sequence-specific DNA binding
6.7e-10 RNA polymerase II cis-regulatory region sequence-specific DNA binding
5.8e-10 Cis-regulatory region sequence-specific DNA binding
1.2e-08 DNA-binding transcription factor activity
1.3e-08 DNA-binding transcription factor activity, RNA polymerase II-specific
6.6e-07 DNA-binding transcription activator activity
4.5e-07 DNA-binding transcription activator activity, RNA polymerase II-specific
3.3e-09 Transcription factor binding
6.6e-08 Protein domain specific binding
1.5e-08 Transferase activity, transferring phosphorus-containing groups
3.9e-08 Kinase activity
1.2e-08 Phosphotransferase activity, alcohol group as acceptor
4.6e-08 Protein kinase activity
8.1e-08 Protein serine/threonine kinase activity
1.5e-07 Protein serine kinase activity
5.8e-10 Chromosome
2.6e-15 Chromatin
1.6e-06 Transcription regulator complex
2.3e-15 Synapse
2.5e-06 Presynapse
9.1e-10 Glutamatergic synapse
9.0e-08 Postsynaptic specialization
1.1e-08 Postsynaptic density
7.9e-09 Asymmetric synapse
9.0e-09 Neuron to neuron synapse
5.1e-10 Postsynapse
9.0e-08 Synaptic membrane
3.1e-06 Postsynaptic membrane
7.9e-09 Dendritic tree
7.9e-09 Dendrite
1.4e-10 Somatodendritic compartment
1.8e-06 Neuronal cell body
1.2e-07 Cell body
6.1e-14 Neuron projection
1.7e-10 Axon
4.2e-04 Thyroid hormone signaling pathway
1.6e-05 Axon guidance
1.9e-04 Hepatocellular carcinoma
2.5e-04 Gastric cancer
2.0e-04 Pathways in cancer
1.9e-04 Proteoglycans in cancer
3.2e-06 MicroRNAs in cancer
2.1e-06 Signaling pathways regulating pluripotency of stem cells
6.6e-04 Insulin signaling pathway
4.0e-04 FoxO signaling pathway
4.0e-04 AMPK signaling pathway
1.8e-04 Longevity regulating pathway
4.0e-04 Growth hormone synthesis, secretion and action
3.7e-05 Endocrine resistance
1.6e-04 Relaxin signaling pathway
1.3e-04 Ras signaling pathway
1.2e-05 MAPK signaling pathway
4.4e-06 Rap1 signaling pathway
6.7e-04 TGF-beta signaling pathway
3.7e-05 Transcriptional misregulation in cancer
B
7.1e-08 Cerebral nuclei
7.5e-09 Subpallium
7.4e-09 Striatum
5.8e-09 Striatum dorsal region
5.8e-09 Caudoputamen
5.3e-09 Striatum (corpus striatum)
5.3e-09 Mantle zone of Str
5.8e-09 Putamen
5.3e-09 Central subpallium (classic basal ganglia)
6.6e-07 Island of Calleja major
5.8e-09 Caudal secondary prosencephalon
5.8e-09 Alar plate of evaginated telencephalic vesicle
5.8e-09 Telencephalic vesicle
1.1e-08 Secondary prosencephalon
1.1e-08 Forebrain
6.6e-07 Ventral group of the dorsal thalamus
7.7e-08 Ventral posterior complex of the thalamus
4.0e-07 Interstitial nucleus of the zona limitans
1.6e-07 Dentate gyrus
3.4e-07 Dentate gyrus molecular layer
1.5e-04 Ventral group of the dorsal thalamus
1.5e-04 Somatosensory areas
2.1e-04 Anterodorsal nucleus
2.0e-05 Brain stem
7.6e-06 Mantle zone of PHyB-I
4.0e-06 Intermediate part of PHyB
7.6e-06 Basal peduncular hypothalamus
1.6e-05 Peduncular (caudal) hypothalamus
6.2e-05 Ventral part of PHyB
6.2e-05 Mantle zone of PHyB-V
1.5e-04 Mammillary body
3.1e-06 Tuberal nucleus
1.5e-04 Medial tuberal nucleus
1.5e-04 Mantle zone of THyB-I
1.5e-04 Intermediate part of THyB
1.8e-04 Dorsal part of THyB
1.7e-04 Mantle zone of THyB-D
3.1e-06 Shell of VMH
1.7e-04 Mantle zone of r1BM
1.1e-04 Medial part of r1B
C
8.9e-60 Hsa-miR-17-5p POSITIVE DOWN target gene
1.1e-64 Hsa-miR-93-5p POSITIVE DOWN target gene
2.3e-56 Hsa-miR-20a-5p POSITIVE DOWN target gene
1.8e-55 Hsa-miR-106b-5p POSITIVE DOWN target gene
1.6e-45 Hsa-miR-106a-5p POSITIVE DOWN target gene
8.0e-56 Hsa-miR-181a-5p POSITIVE DOWN target gene
4.7e-150 Hsa-miR-27b-3p POSITIVE DOWN target gene
8.0e-159 Hsa-miR-128-3p POSITIVE DOWN target gene
3.8e-42 Hsa-miR-30b-5p POSITIVE DOWN target gene
9.4e-50 Hsa-miR-30c-5p POSITIVE DOWN target gene
6.3e-56 Hsa-miR-30e-5p POSITIVE DOWN target gene
4.4e-55 Hsa-miR-30d-5p POSITIVE DOWN target gene
7.4e-46 Hsa-miR-30a-5p POSITIVE DOWN target gene
1.2e-41 Hsa-miR-19a-3p POSITIVE DOWN target gene
6.9e-53 Hsa-miR-26a-5p POSITIVE DOWN target gene
9.9e-39 Hsa-miR-101-3p POSITIVE DOWN target gene
4.1e-40 Hsa-miR-146a-3p POSITIVE DOWN target gene
8.0e-43 Hsa-miR-130a-3p POSITIVE DOWN target gene
2.0e-40 Hsa-miR-92a-3p POSITIVE DOWN target gene
1.8e-40 Hsa-miR-32-5p POSITIVE DOWN target gene
0.0e+00 MiR-27-3p target gene
0.0e+00 MiR-27 target gene
0.0e+00 MiR-128 target gene
0.0e+00 MiR-128-3p target gene
2.8e-102 MiR-30-5p target gene
7.2e-94 MiR-30 target gene
1.4e-90 MiR-30-5p/384-5p target gene
1.2e-95 MiR-181-5p target gene
4.8e-96 MiR-181 target gene
2.2e-89 MiR-182 target gene
5.0e-87 MiR-182-5p target gene
3.5e-88 MiR-9-5p target gene
5.0e-89 MiR-9 target gene
7.1e-137 MiR-144-3p target gene
4.2e-140 MiR-144 target gene
9.6e-91 MiR-101 target gene
1.0e-92 MiR-101-3p target gene
5.5e-87 MiR-101-3p.1 target gene
7.0e-100 MiR-218 target gene
6.5e-100 MiR-218-5p target gene
D
2.6e-10 TP63 CHEA
1.7e-18 AR CHEA
3.4e-18 SMAD4 CHEA
1.7e-12 NFE2L2 CHEA
3.0e-11 GATA2 CHEA
1.7e-12 GATA1 CHEA
8.8e-07 RUNX1 CHEA
3.0e-14 REST CHEA
2.3e-30 SUZ12 CHEA
1.9e-09 NANOG CHEA
3.2e-17 TCF3 CHEA
1.2e-14 SOX2 CHEA
6.0e-11 SALL4 CHEA
7.8e-14 TCF3 ENCODE
3.6e-44 UBTF ENCODE
3.6e-06 CTCF ENCODE
1.5e-05 CHD1 ENCODE
4.9e-07 RCOR1 ENCODE
2.6e-06 CREB1 CHEA
3.6e-06 PBX3 ENCODE
1.7e-02 MEF2C target gene
6.4e-05 IKZF1 target gene
4.4e-02 TFAP2A target gene
4.4e-02 ESR1 target gene
4.5e-02 ZNF217 target gene
4.6e-02 NR2C2 target gene
5.0e-08 NCOR1 target gene
3.9e-03 GTF3C2 target gene
1.2e-03 PRDM1 target gene
2.4e-03 CEBPZ target gene
1.4e-02 MYB target gene
1.1e-03 CBX2 target gene
4.7e-07 CBX8 target gene

Figure S2. Functional annotation analysis of the miR-128-3p target genes.
A hierarchical clustering tree of gene ontology pathways (A), sub-brain localization (B), co-targeting (C), and transcription factor network (D) enrichment analysis generated by ShinyGO for the miR-128-3p target genes (n=1225). Larger dots indicate more significant p-values. FDR cutoff >0.05.

A Gene Ontology: Biological Processes, Molecular Function, Cellular Compartment and KEGG pathways [13] (Table S1/Spreadsheet: 7-10).

B Up-regulation (left) and down-regulation (right) of miR-128-3p target genes within sub-brain localizations [19] (Table S1/Spreadsheet: 11-12).

C Targets of miR-128-3p by TargetScan analysis [16] and inverse correlation of miR-128-3p targets by Diana web server analysis [20] (Table S1/Spreadsheet: 13-14).

D Targets of miR-128 co-targeted by transcription factors by Enrichr (gene set enrichment analysis tool) [17] and ENCODE (Encyclopaedia of DNA Elements) portal [21] (Table S1/Spreadsheet: 15-16).

Table S1.

Spreadsheet 1: miEAA - miRNA Enrichment and Annotation – Full analysis results
Spreadsheet 2: dataset pertaining to the Figure S1A – total dataset
Spreadsheet 3: dataset pertaining to the Figure S1A – dataset upon cutoff
Spreadsheet 4: dataset pertaining to the Figure S1B
Spreadsheet 5: dataset pertaining to the Figure S1C
Spreadsheet 6: TargetScan 8.0 – list of hsa-miR-128-3p targets
Spreadsheet 7: dataset pertaining to the Figure S2A - upper left
Spreadsheet 8: dataset pertaining to the Figure S2A - upper right
Spreadsheet 9: dataset pertaining to the Figure S2A - bottom left
Spreadsheet 10: dataset pertaining to the Figure S2A - bottom right
Spreadsheet 11: dataset pertaining to the Figure S2B - left
Spreadsheet 12: dataset pertaining to the Figure S2B - right
Spreadsheet 13: dataset pertaining to the Figure S2C - left
Spreadsheet 14: dataset pertaining to the Figure S2C - right
Spreadsheet 15: dataset pertaining to the Figure S2D - left
Spreadsheet 16: dataset pertaining to the Figure S2D - right

SUPPLEMENTAL REFERENCES

1. Keller, A., et al., *miRNATissueAtlas2: an update to the human miRNA tissue atlas.* Nucleic Acids Res, 2022. **50**(D1): p. D211-D221.
2. Fehlmann, T., E. Meese, and A. Keller, *Exploring ncRNAs in Alzheimer's disease by miRMaster.* Oncotarget, 2017. **8**(3): p. 3771-3772.
3. Fehlmann, T., et al., *Web-based NGS data analysis using miRMaster: a large-scale meta-analysis of human miRNAs.* Nucleic Acids Res, 2017. **45**(15): p. 8731-8744.
4. Fehlmann, T., et al., *miRMaster 2.0: multi-species non-coding RNA sequencing analyses at scale.* Nucleic Acids Res, 2021. **49**(W1): p. W397-W408.
5. Kozomara, A., M. Birgaoanu, and S. Griffiths-Jones, *miRBase: from microRNA sequences to function.* Nucleic Acids Res, 2019. **47**(D1): p. D155-D162.
6. Guttman, M., et al., *Chromatin signature reveals over a thousand highly conserved large non-coding RNAs in mammals.* Nature, 2009. **458**(7235): p. 223-7.
7. Consortium, R.N., *RNAcentral 2021: secondary structure integration, improved sequence search and new member databases.* Nucleic Acids Res, 2021. **49**(D1): p. D212-D220.
8. Chan, P.P. and T.M. Lowe, *GtRNAdb 2.0: an expanded database of transfer RNA genes identified in complete and draft genomes.* Nucleic Acids Res, 2016. **44**(D1): p. D184-9.

9. Fang, S., et al., *NONCODEV5: a comprehensive annotation database for long non-coding RNAs.* Nucleic Acids Res, 2018. **46**(D1): p. D308-D314.
10. Aparicio-Puerta, E., et al., *miEAA 2023: updates, new functional microRNA sets and improved enrichment visualizations.* Nucleic Acids Res, 2023. **51**(W1): p. W319-W325.
11. Ge, S.X., D. Jung, and R. Yao, *ShinyGO: a graphical gene-set enrichment tool for animals and plants.* Bioinformatics, 2020. **36**(8): p. 2628-2629.
12. Jin, Z., et al., *KEGG tools for classification and analysis of viral proteins.* Protein Sci, 2023. **32**(12): p. e4820.
13. Thomas, P.D., et al., *PANTHER: Making genome-scale phylogenetics accessible to all.* Protein Sci, 2022. **31**(1): p. 8-22.
14. Hawrylycz, M.J., et al., *An anatomically comprehensive atlas of the adult human brain transcriptome.* Nature, 2012. **489**(7416): p. 391-399.
15. Paraskevopoulou, M.D., et al., *DIANA-microT web server v5.0: service integration into miRNA functional analysis workflows.* Nucleic Acids Res, 2013. **41**(Web Server issue): p. W169-73.
16. Agarwal, V., et al., *Predicting effective microRNA target sites in mammalian mRNAs.* Elife, 2015. **4**.
17. Kuleshov, M.V., et al., *Enrichr: a comprehensive gene set enrichment analysis web server 2016 update.* Nucleic Acids Res, 2016. **44**(W1): p. W90-7.
18. Consortium, E.P., *An integrated encyclopedia of DNA elements in the human genome.* Nature, 2012. **489**(7414): p. 57-74.
19. Ding, S.L., et al., *Comprehensive cellular-resolution atlas of the adult human brain.* J Comp Neurol, 2016. **524**(16): p. 3127-481.
20. Maragkakis, M., et al., *DIANA-microT Web server upgrade supports Fly and Worm miRNA target prediction and bibliographic miRNA to disease association.* Nucleic Acids Res, 2011. **39**(Web Server issue): p. W145-8.
21. Luo, Y., et al., *New developments on the Encyclopedia of DNA Elements (ENCODE) data portal.* Nucleic Acids Res, 2020. **48**(D1): p. D882-D889.
